# Divergent Population Structure in Five Common Rockfish Species of Puget Sound, WA Suggests the Need for Species‐Specific Management

**DOI:** 10.1111/mec.17590

**Published:** 2024-11-25

**Authors:** Anita Wray, Eleni Petrou, Krista M. Nichols, Robert Pacunski, Larry LeClair, Kelly S. Andrews, Dana Haggarty, Lorenz Hauser

**Affiliations:** ^1^ School of Aquatic and Fishery Sciences University of Washington Seattle Washington USA; ^2^ Conservation Biology Division, Northwest Fisheries Science Center National Marine Fisheries Service, NOAA Seattle Washington USA; ^3^ Washington Department of Fish and Wildlife Olympia Washington USA; ^4^ Pacific Biological Station Fisheries and Oceans Canada Nanaimo British Columbia Canada; ^5^ Biology Department University of Victoria Victoria Canada; ^6^ Zoology Department Nelson Mandela University Gqeberha South Africa

**Keywords:** conservation genetics, fisheries management, population dynamics, RADseq, *Sebastes*

## Abstract

Quantifying connectivity between endangered or threatened marine populations is critical information for management and conservation, especially where abundance and productivity differ among such populations. Spatial patterns of such connectivity depend not only on extrinsic factors such as oceanography and bathymetry but also on intrinsic species‐specific factors such as life history, demography and the location of glacial refugia. Nevertheless, population structure is often inferred from related or ecologically similar species. For example, the population structure in most rockfish species (*Sebastes* spp.) in the Salish Sea and the US West Coast is currently inferred from genetic data of three species that are known to hybridise in Puget Sound. Here, we determined the population structure and connectivity in five Puget Sound Rockfish species (Black [*Sebastes melanops*], Yellowtail [*S. flavidus*], Redstripe [*S. proriger*], Greenstriped [*S. elongatus*], and Puget Sound Rockfish [*S. emphaeus*]) from over 12,000 restriction‐site associated DNA sequencing (RADseq) loci. We found species‐specific patterns of genetic differentiation, attributable to both extrinsic and intrinsic factors. Specifically, Black and Puget Sound rockfishes showed no genetic differentiation; Yellowtail and Greenstriped rockfishes were structured according to known geographic barriers; and Redstripe Rockfish revealed evidence for temporal genetic differentiation, suggesting irregular recruitment influences population structure. Only Yellowtail Rockfish followed the federal DPS boundaries generally assumed for rockfish, further emphasizing the importance of species‐specific management for the effective recovery and management of these rockfish populations and of marine species in general.

## 1 Introduction

While many marine species are characterised by large population sizes and high levels of connectivity, there are sharp genetic discontinuities in many species (Hauser and Carvalho 2008). Such phylogeographic breaks often develop where past or present barriers to dispersal reduce the homogenising effects of gene flow (Pelc, Warner, and Gaines 2009) or where previously isolated populations come into secondary contact (Johannesson et al. 2020), often after expansion from different glacial refugia (Smith et al. 2022). Phylogeographic breaks that coincide in several different species are particularly interesting as they provide an opportunity to distinguish between the effects of extrinsic environment context and intrinsic species‐specific factors such as the underlying genomic architecture, life history, ecology, and glacial history (Johannesson et al. 2020). Such multispecies phylogeographic breaks often coincide with biogeographic breaks separating different species assemblages and are commonplace in the oceans around the world (Bowen et al. 2016), such as the Baltic/North Sea (Geburzi et al. 2022), Mediterranean Sea/Atlantic (Patarnello, Volckaert, and Castilho 2007), in South African biogeographic regions (Teske et al. 2011) and Point Conception in California (Sivasundar and Palumbi 2010).

Intraspecific genetic boundaries are also highly relevant for the conservation and management of marine species, not only because they provide clear boundaries between management units (Hauser and Carvalho 2008) but also because they describe the distribution of genetic diversity within species (Bowen et al. 2016) and may represent boundaries to range shifts caused by environmental change and so cause population extirpation at the trailing edge of a distribution shift (Pinsky, Selden, and Kitchel 2020). Such trailing edge populations are of high conservation concern because they may be valuable sources of genetic variability allowing adaptation to climate change in larger core populations (Fisher et al. 2022). The mechanisms leading to the development and maintenance of phylogeographic breaks are therefore crucial for both short‐term management and longer‐term conservation. Nevertheless, such data are not commonly available, especially in a comparison of several closely related species, which may allow consideration of both species‐specific and environment‐specific factors.

Marine populations in the northeast Pacific show strong genetic signals of past glaciations that affected large proportions of the habitat but also left several glacial refugia (Shafer et al. 2010). Especially in regions where there are bathymetric or oceanographic barriers, genetic differentiation between populations is still very apparent and likely predates the last glaciation (Canino et al. 2010; Grant and Cheng 2012; Liu et al. 2012; Petrou et al. 2013; Grant and Bringloe 2020). Such bathymetric and oceanographic barriers are especially prominent in major coastal inlets such as the Salish Sea at the Canada/US border, with its largest estuary, the Puget Sound. The Salish Sea was completely covered in ice during the last glacial maximum about 17,000 years ago (kya) (Mann and Gaglioti 2024), but ice‐free glacial refugia likely persisted to the north of the Salish Sea at the northwest coast of Vancouver Island (Hebda et al. 2022) and between Vancouver Island and Haida Gwaii (Shaw et al. 2020). Refugial populations of marine species may have subsequently colonised the Salish Sea and Puget Sound, where they remained isolated by narrow straits with a series of shallow sills that affect oceanographic patterns and limit the dispersal of planktonic life history stages (Engie and Klinger 2007). In addition, differences in genome structure, in particular, chromosome inversions, may maintain isolation even if there is some gene flow (Petrou et al. 2021). As a result, many species have distinct coastal and Puget Sound populations, for example, Yelloweye Rockfish (*Sebastes ruberrimus*) (Andrews et al. 2018), Pacific Cod (*Gadus macrocephalus*) (Canino et al. 2010), Pacific hake (*Merluccius productus*) (Iwamoto, Ford, and Gustafson 2004), lingcod (*Ophiodon elongatus*) (Longo et al. 2020) and Dungeness crab (*Cancer magister*) (Jackson and O'Malley 2017).

**FIGURE 1:**
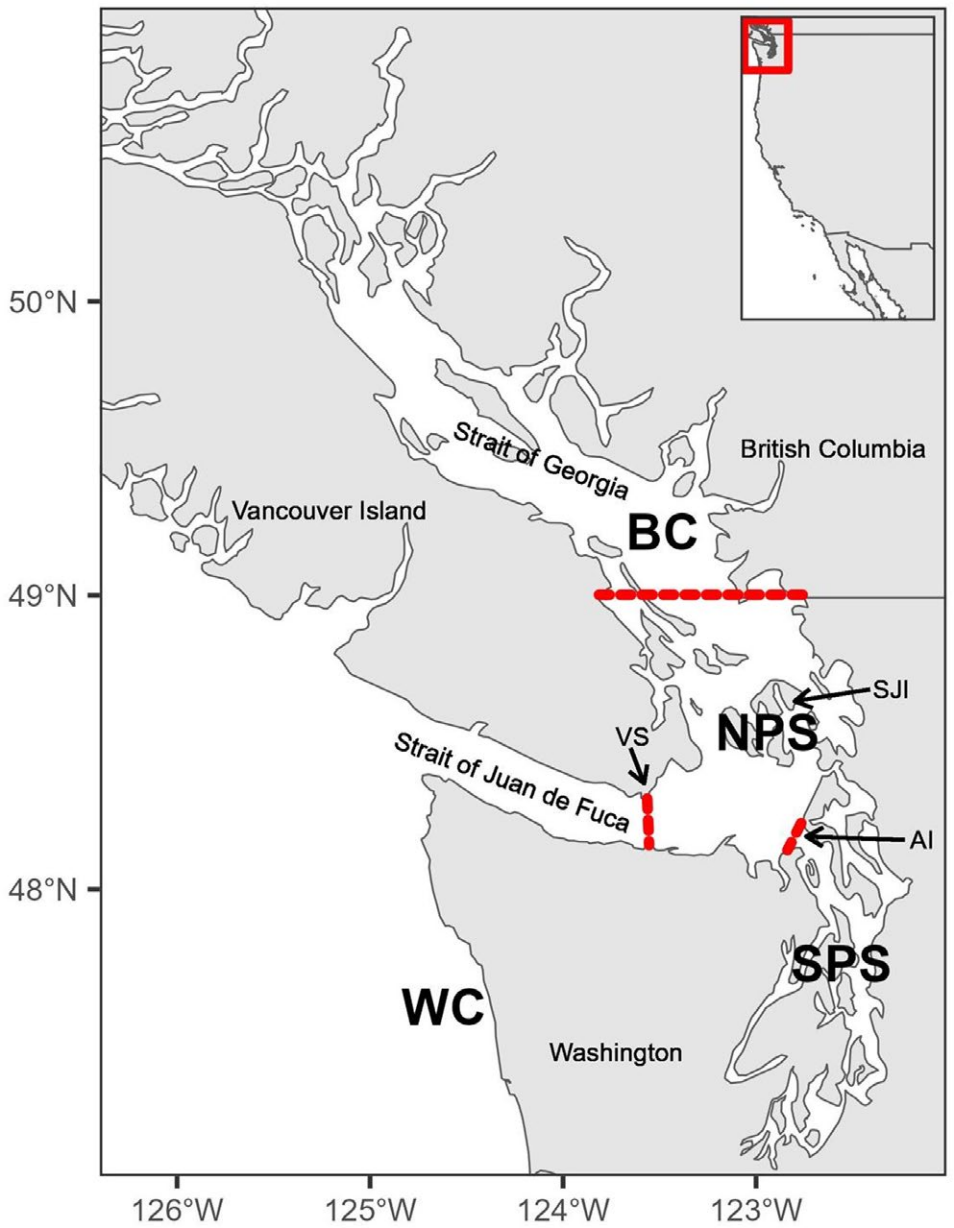
Map showing the sampling regions defined in this study. Sampling areas are north Puget Sound (NPS), south Puget Sound (SPS), British Columbia (BC), and the Washington Coast (WC) and are separated by the red lines for the purpose of this study. Victoria Sill (VS), San Juan Islands (SJI) and Admiralty Inlet (AI) are known biogeographic barriers in the region and were used to define the sampling areas.

While genetic differentiation is common in Salish Sea marine populations, the extent of this differentiation and the distribution of subpopulations vary considerably between species. For example, genetic differentiation is relatively weak (*F*_ST_ < 0.010) in lingcod (Longo et al. 2020) and Dungeness crab (Jackson and O'Malley 2017) but stronger (*F*_ST_ > 0.015) in Pacific cod (Drinan et al. 2018) and Yelloweye Rockfish (Andrews et al. 2018). Population boundaries are situated on several different shallow sills (Victoria Sill, Admiralty Inlet, San Juan Islands, Figure 1) and often do not coincide between species. In fact, relatively minor differences in life history can determine whether there is a population structure or not (Andrews et al. 2018, 2021). Nevertheless, such boundaries are often inferred from data of related species and used for conservation and management (Drake et al. 2010) – often with undesirable outcomes, such as the recent reversal of the listing decision of the Puget Sound Canary Rockfish (*Sebastes pinniger*) Distinct Population Segment (DPS) under the US Endangered Species Act because the previously assumed population boundary could not be confirmed by new genetic data (Andrews et al. 2018). Species‐specific data are therefore needed both for a thorough understanding of population structure and for conservation and management.

Pacific rockfishes (*Sebastes* spp.) are an ideal group to address these questions. There are over 100 recognised species that display common life history characteristics such as long lifespans, late maturity, slow growth, viviparity, high fecundity, relatively long larval duration (3–6 months), and sporadic recruitment (Love, Yoklavich, and Thorsteinson 2002). Nevertheless, they differ in adult habitat, spawn timing, larval duration, and other life history characteristics that may affect dispersal and population connectivity, and differences in their contemporary distribution suggest different demographic and genetic dynamics during Pleistocene glaciations. Connectivity among rockfish populations is also of more immediate management and conservation interest, as their specific life histories make them particularly vulnerable to exploitation and local population depletion. Indeed, of the 67 species of rockfishes managed under the Pacific Coast Groundfish Fishery Management Plan, 48 species are considered moderately or highly vulnerable to overfishing (Pacific Fishery Management Council 2020). After overfishing in the 1990s and the implementation of strict rebuilding plans, all but one species (Yelloweye Rockfish) are now considered rebuilt along the West Coast of North America (Pacific Fishery Management Council 2020). Nevertheless, similar measures in the Salish Sea have not led to recovery (Williams, Levin, and Palsson 2010), and recreational catch‐per‐unit‐effort data suggest a 3.8% annual decline of total rockfish abundance (Tolimieri et al. 2017). Connectivity between the Salish Sea and coastal populations is therefore unknown for most species, leading to considerable uncertainty in management and conservation.

The objective of this study was to compare population structure among five common rockfish species in the Salish Sea from over 10,000 genome‐wide markers obtained by restriction site‐associated DNA sequencing (RADseq) (Baird et al. 2008). These markers provided not only extremely powerful species identification and population analyses without the need for expensive and laborious marker development but also allowed the detection of chromosome inversions and signatures of selection. The five species were chosen because they are the most common in the area, except for Brown (*S. auriculatus*), Copper (*S. caurinus*) and Quillback (*S. maliger*) Rockfish, which are known to hybridise and are the subject of another study (Wray et al. 2024). The five species belong to three morphologically defined subgenera (Kendall and Arthur 2000), which also represent different evolutionary clades (Hyde and Vetter 2007): *Sebastosmus* (Yellowtail [*S. flavidus*] and Black [*S. melanops*] Rockfish), *Allosebastes* (Puget Sound [*S. emphaeus*] and Redstripe [*S. proriger*] Rockfish) and *Hispanicus* (Greenstriped *[S. elongatus*] Rockfish). More importantly, the species show subtle differences in timing of parturition, larval pelagic duration, adult habitat, and site fidelity. Specifically, we had the following aims:

- To determine the extent of population structure in relation to life history and genome structure. Our expectation was that species with less dispersal (shorter larval duration, parturition in winter when water tends to be retained in the Salish Sea (Andrews et al. 2021)), deeper adult habitat, higher site fidelity (more effective separation by habitat and shallow sills) and large chromosome inversions would have more extensive population structure.
- To identify the location of phylogeographic boundaries in species with significant population structure. The best‐known boundary is the Victoria sill (Drinan et al. 2018), which separates the Salish Sea from the coast, although the Admiralty Inlet separating Puget Sound (Drake et al. 2010), or the San Juan Islands separating the Georgia Basin from the Strait of Juan de Fuca may also represent barriers.
- To establish the extent of dispersal and gene flow across those boundaries—even if phylogeographic boundaries exist, some dispersal may occur as for example in Pacific cod (Drinan et al. 2018; Fisher et al. 2022). If so, we would expect to find individuals outside the geographic distribution of their genetic cluster.
- To detect hybridisation and introgression resulting in gene flow from individuals belonging to two or more genetic clusters.

## 2 Materials and Methods

### 2.1 Sampling

We used samples from 279 individuals from five species of rockfish (Black, Yellowtail, Redstripe, Greenstriped, and Puget Sound, Figure 1, Table 2) that were collected in 1999–2021 by the Washington Department of Fish and Wildlife (WDFW), the US National Marine Fisheries Service (NOAA NMFS) and the Department of Fisheries and Oceans (DFO Canada). Individual fin clips were preserved in 95% ethanol or dried on Whatman filter paper. Individuals were collected from four regions: (1) southern Puget Sound (Puget Sound proper, south of Admiralty Inlet, SPS), (2) northern Puget Sound (US Salish Sea north of Admiralty Inlet, and east of the Victoria Sill, NPS), (3) British Columbia (Canadian Salish Sea north of the US/Canada border, BC), and (4) the US West Coast (US Pacific Coast west of Victoria Sill, WC). Due to differences in the abundance and distribution of species across this geographic range, sample sizes varied between regions, and we have no Puget Sound Rockfish from WC and one Greenstriped Rockfish from NPS.

### 2.2 DNA Extraction, Library Preparation and Sequencing

Genomic DNA was extracted using the Nexttec DNA isolation kit (Nexttec Incorporated, Middlebury, VT, USA) following the manufacturer's protocol and quantified using a Qubit Fluorometer (ThermoFisher Scientific, Waltham, MA, USA). DNA concentration was normalised to 125 ng in 10 μL of molecular‐grade water. Restriction site‐associated DNA sequencing (RADseq) libraries were prepared using a version of the Ali, Jeffres, and Miller (2016) protocol without the targeted bait capture step, referred to in the literature as BestRAD (https://github.com/merlab‐uw/Protocols/blob/main/bestRAD). Briefly, genomic DNA was digested using the *Sbf*I enzyme. An adapter (P1) containing a forward amplification primer site, an Illumina sequencing primer site, and an individual 6 bp barcode was ligated to each fragment at the restriction site end. Fragments were then randomly sheared using sonication and size‐selected to 300–500 bp in length. Subsequently, P2 adapters were ligated to the reverse end and libraries were amplified by PCR. Each library was assessed for quality on a 1% agarose gel and a Bioanalyzer DNA 1000 kit (Agilent Technologies, Santa Clara, CA). Libraries were pooled in equimolar amounts and sequenced on a NovaSeq (paired end, 116 bp or 150 bp) at the University of Oregon, either an S4 or SP run type. Ninety‐six individuals were randomly included in one of six RADseq libraries to avoid any lane effect (Leigh et al. 2018).

### 2.3 Initial Filtering

Raw sequence data were quality‐checked using *MultiQC* (Ewels et al. 2016). Prior to SNP calling and genome alignment, raw sequences were demultiplexed using *process_radtags* in the *Stacks* v2.60 pipeline (Catchen et al. 2011; Rochette, Rivera‐Colón, and Catchen 2019). Sequences were trimmed to 104 bases and filtered for quality. Individuals with fewer than 250,000 total reads were excluded from downstream analysis (Krohn et al. 2018). Our paired‐end sequences were then aligned to the Honeycomb Rockfish (*S. umbrosus*) genome from GenBank (NCBI Accession Number: PRJNA562243) with *Bowtie2* v2.4. using the ‘very‐sensitive’ option (Langmead and Salzberg 2012). The Honeycomb Rockfish genome is one of only two annotated full genomes and was chosen due to its closer phylogenetic relationship to our focal species (Hyde and Vetter 2007). Following genome alignment, SNP calling and basic population genetics statistics were calculated using the *gstacks* (*marukilow* model) and *populations* modules from the *Stacks* pipeline. SNPs were called if they had a minimum mapping quality of 40.

### 2.4 Misidentification Analysis

To identify any cases of (1) misidentification of species during field sampling or (2) interspecific hybridisation, raw sequences for all individuals were analysed together for eight species (five from this study and Brown, Quillback, and Copper Rockfish from Wray et al. 2024) immediately after genome alignment. SNP calling and basic population genetics statistics were calculated using the *gstacks* and *populations* modules from the *Stacks* pipeline. SNPs were filtered in *VCFtools* v0.1.13 (Danecek et al. 2011) following published recommendations (O'Leary et al. 2018) requiring that loci meet the following criteria: minimum genotype depth ≥ 5, mean minimum read depth ≥ 15, genotype call rate ≥ 80% (*−minDP 5*, *−min‐meanDP 15*, *−max‐missing 0.80*). Additionally, we avoided SNPs with fixed differences between species because they would likely reveal differences between only two species. Therefore, we chose the first SNP on each RADtag using the –write‐single‐snp option in *populations*. Additionally, we did not filter for HWE because a reduction of heterozygosity due to species‐specific (subpopulation) structure would likely influence HWE *p*‐values due to the Wahlund effect. We plotted all eight species together in a principal components analysis (PCA). Any individuals that visually grouped with a species different than their field identification were considered misidentification and removed from downstream analysis.

### 2.5 Species‐Specific Analyses

SNPs were filtered following published recommendations (O'Leary et al. 2018) requiring that loci meet the following criteria: minimum genotype depth ≥ 5, mean minimum read depth ≥ 15, and genotype call rate ≥ 80%. In contrast to the interspecific analyses, however, we chose the SNP with the highest minor allele frequency on each RADtag. SNPs with genotype frequencies that were significantly different than expectations under Hardy–Weinberg Equilibrium (HWE) were also removed using the following procedure: locus‐specific *p*‐values were calculated across individuals for each region using the exact test within the R package *pegas* v1.1 (Paradis 2010). *P*‐values were then combined across individuals for each locus using Fisher's combination of probabilities and adjusted to *q*‐values for the false discovery rate (Benjamini and Hochberg 1995). Loci with *q*‐values below 0.05 were considered significantly out of HWE and removed from downstream analysis. Summary statistics were calculated using *VCFtools* v0.1.13 (individual read depth) (Danecek et al. 2011) and *hierfstat* v0.5–11 (*H*_O_, *H*E, *F*_IS_) (Goudet 2005).

Patterns of genetic population structure were determined with PCA, *STRUCTURE* analyses, and by estimating pairwise *F*_ST_. After removing misidentified individuals, we used the R package *adegenet* v2.1.8 (Jombart 2008) to compute a PCA. To investigate any patterns unexplained by geographic region, we re‐coloured each individual within the PCA graph according to collection year, sex, length, depth caught, read depth, or DNA quantity.

In addition, we used *STRUCTURE* v2.3.4 (Pritchard, Stephens, and Donnelly 2000) to estimate the most likely genetic clustering pattern across individuals and to identify hybrids between genetic clusters. *STRUCTURE* was run without a priori population knowledge and using the admixture model. Two replicates were run for 1–10 clusters with a burn‐in of 10,000 iterations and 100,000 MCMC reps. We used the ΔK statistic (Evanno, Regnaut, and Goudet 2005) and the mean *L*(*K*) from *Structure Harvester* (Earl and vonHoldt 2012) to estimate the number of clusters *K*.

Population boundaries were identified by plotting pie charts of the average *STRUCTURE* cluster memberships across all individuals in a sample onto a map of the area. Areas of sharp changes in cluster membership were correlated with known bathymetric and oceanographic features of the Salish Sea. Dispersal across these boundaries was determined from individuals collected outside the distribution of their cluster as determined by *STRUCTURE* and PCA. Gene flow across boundaries was identified from individuals that belonged to two or more *STRUCTURE* clusters or that had an intermediate position in the PCA.

Overall and pairwise *F*_ST_ values (Weir and Cockerham 1984) were estimated with the R package *hierfstat* v0.5–11 (Goudet 2005) both among geographic regions and among genetic clusters as identified by *STRUCTURE*. *F*_ST_ values were considered significant if the 95% confidence interval obtained from 1000 bootstrap iterations did not include zero.

Evidence for chromosome inversions was detected by estimating linkage disequilibrium within each chromosome using *PLINK* v1.07 (Purcell et al. 2007). *R* ^2^ values were then mapped on each chromosome to identify blocks of highly linked loci using the R function *LDheatmap* v1.0–6 (Shin et al. 2006). Regions of chromosomes with loci in strong LD (*r* ^2^ > 0.5) over extended blocks (distance > 1 Mb) were analysed using PCAs in *adegenet* v2.1.8 (Jombart 2008) to determine whether individuals clustered in the three‐stripe patterns consistent with chromosomal inversions (Hoffmann and Rieseberg 2008; Petrou et al. 2021).

To test for sweepstake recruitment (Hedgecock and Pudovkin 2011) and identify related individuals (% IBD > 10%), genetic relatedness was calculated on all pairs of individuals with *PLINK* v1.07 (Purcell et al. 2007). Effective population size was estimated using the linkage disequilibrium model in *NeEstimator* (Do et al. 2014) with a minor allele frequency (MAF) cut‐off of 0.05. *N*_e_ estimates were adjusted for limited genome size using the method of Waples, Larson, and Waples (2016). Manhattan plots of per‐locus *F*_ST_ were calculated using *hierfstat* v0.5–11 (Goudet 2005) to test for loci under selection.

Nucleotide diversity (*π*) and *d*_xy_ were calculated on a 10 kb window with *pixy* v1.2.7.beta1 (Korunes and Samuk 2021) using an all‐site dataset, which included all called SNPs and invariant sites. The all‐site dataset was created using *BCFtools* (Danecek et al. 2021) and filtered using *VCFtools* (Danecek et al. 2011) according to Hirao et al. (2024) (*remove‐indels, minDP 20, minQ 30, max‐missing 0.7, max‐alleles 2, and minMQ 30 for SNPs; max‐maf 0, minDP 20, max‐missing 0.7, and minMQ 30 for invariant sites*). Net nucleotide diversity within (π) and between (*d*_*xy*_) genetic clusters identified by *STRUCTURE* was calculated using the formula $d_{a}=d_{\mathrm{xy}}-\frac{\left(\pi_{x}+\pi_{y}\right)}{2}$ (Nei 1987) and used to estimate divergence time using the formula $T=d_{a}/2\lambda$ (Nei 1987) assuming a mutation rate (λ) of 5.3 × 10^−9^/bp × generation which appears to be relatively consistent among teleost species (Bergeron et al. 2023; Zhang 2023) and was adjusted to a per year rate using generation times from Kolora et al. (2021).

## 3 Results

### 3.1 Misidentification Analysis

In total, 14 individuals were removed due to misidentification. Visual discrimination using a PCA suggested that these individuals were not hybrids, but misidentification in the field (Figure S1). All other individuals included in the species‐specific analyses visually grouped with their own species, suggesting that there was no hybridisation or contamination between species.

### 3.2 Species‐Specific Analyses

**FIGURE 2:**
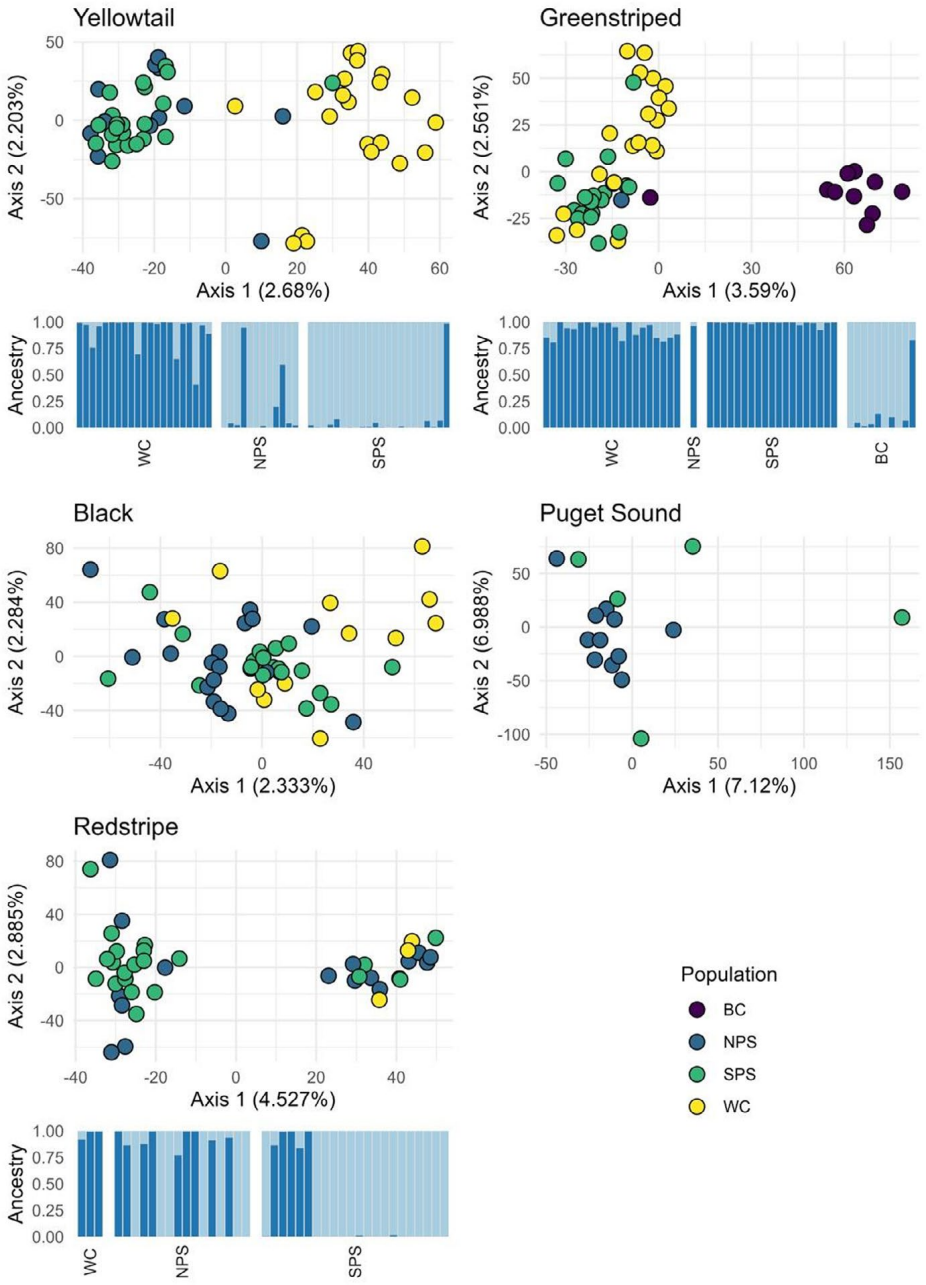
PCA and *STRUCTURE* plots of five rockfish species suggest different structuring patterns. For the *STRUCTURE* plots, each bar represents an individual, and the colour represents the genetic cluster to which each fish was assigned. Regions are ordered from the coast (WC), into Puget Sound from the north (NPS) to the south (SPS) and British Columbia (BC). Individuals within each location are ordered from south to north. For the PCAs, each point represents an individual fish, coloured by their sampling location. There are no *STRUCTURE* plots for Puget Sound and Black Rockfish because only one cluster was discovered.

For **Yellowtail Rockfish**, 18,979 loci were retained after filtering. Three individuals were removed from analysis two because of low read count and one because of field misidentification (Figure S1). PCA and *STRUCTURE* plots revealed WC as a separate population from NPS and SPS (Figure 2) and identified two individuals with WC ancestry in Puget Sound and six individuals with ancestry from both Puget Sound and the Washington Coast (*Q* ≤ 0.95). *F*_ST_ estimates were non‐significant for the NPS‐SPS comparison but small and significant for the SPS‐WC and NPS‐WC comparisons (Table 1). We found no chromosomes with highly linked regions, suggesting that there were no large chromosomal inversions. Loci with high *F*_ST_ were distributed across the chromosome (Figure S2). Time since divergence calculated from the all‐sites VCF file between WC and NPS/SPS was estimated to be 18,978 years (Table S1).

**TABLE 1:** Pairwise Weir and Cockerham *F*_ST_ estimates for five species of rockfish in Puget Sound and the Washington Coast.

	Yellowtail		Greenstriped			Black		Puget Sound	Redstripe	
	NPS	WC	NPS	WC	BC	NPS	WC	NPS	NPS	WC
SPS	0	**0.008**	*0*	**0.003**	**0.025**	**0.001**	**0.001**	0.002	**0.002**	**0.012**
NPS		**0.006**		*0.003*	***0.024***		**0.001**			0
WC					**0.021**					

*Note:* Bolded estimates are significantly greater than zero based on a permutation test. Italicised estimates are pairwise comparisons involving a single Greenstriped Rockfish from NPS.

Our **Greenstriped Rockfish** dataset retained 14,439 loci after filtering. Four individuals were removed from analysis, two because of low read count and two because of field misidentification. Both misidentified individuals clustered with Redstripe Rockfish (Figure S1). Only one individual from NPS was included in our final dataset, limiting our ability to make conclusions about connectivity throughout this geographic region (Table 2). The PCA separated individuals collected in British Columbia from all other individuals along PC1, while PC2 showed some separation of WC individuals from SPS individuals (Figure 2). All British Columbia individuals were collected in 2021 (Figure S3) but were not a significantly different size (Figure S4). *STRUCTURE* results suggest similar ancestry for the WC, NPS, and SPS regions and separate ancestry for the BC region. Similar distinctions were observed in the pairwise *F*_ST_ analysis in which BC showed large and significantly different estimates with all other regions (*F*_ST_ = 0.021–0.025, Table 1). Differentiation between the WC region and the SPS region (*F*_ST_ = 0.003) was significant, but approximately 10 times smaller. Our linkage disequilibrium results located one block of highly linked loci on Chromosome 16 (Figure S5). The PCA of the highly linked region was split into two groups highly correlated to sex (Figure S6). Additionally, heterozygosity in this region was high for males, and very low for females (Figure S7), suggesting an XY sex‐determining region. The whole genome PCA (Figure 2) did not change whether this region was included or not. Divergence time calculated from the all‐sites VCF file between BC and NPS/SPS/WC individuals was estimated to be approximately 41,097 years (Table S1).

**TABLE 2:** Summary statistics for five species of rockfish in Puget Sound, the Washington Coast, and British Columbia.

		SPS	NPS	WC	BC
Yellowtail	*N*	22	12	21	
	*H*_O_	0.27	0.27	0.27	
	*H*_E_	0.28	0.28	0.28	
	*F*_IS_	0.026	0.045	0.038	
	*π*	0.0017	0.0017	0.0017	
Greenstriped	*N*	19	1	20	10
	*H*_O_	0.28	0.28	0.28	0.26
	*H*_E_	0.29	—	0.29	0.28
	*F*_IS_	0.022	—	0.022	0.045
	*π*	0.0013	0.0015	0.0014	0.0013
Black	*N*	21	18	12	
	*H*_O_	0.28	0.26	0.25	
	*H*_E_	0.27	0.27	0.27	
	*F*_IS_	−0.03	0.02	0.06	
	*π*	0.0015	0.0015	0.0015	
Puget Sound	*N*	5	11		
	*H*_O_	0.26	0.26		
	*H*_E_	0.27	0.26		
	*F*_*I*S_	0.025	0.017		
	*π*	0.0017	0.0017		
Redstripe	*N*	22	16	3	
	*H*_O_	0.30	0.29	0.29	
	*H*_E_	0.31	0.31	0.30	
	*F*_IS_	0.032	0.039	0.05	
	*π*	0.0015	0.0015	0.0014	

*Note:* All values were calculated using the R package *hierfstat* v0.5–11 (*H*_O_, *H*_E_, *F*_IS_) (Goudet 2005) and *pixy* v1.2.7.beta1 (π) (Korunes and Samuk 2021). Nucleotide diversity (*π*) was calculated using an all‐site dataset, which included all called SNPs and invariant sites.

Abbreviations: *F*_IS_, inbreeding coefficient; *H*_E_, average expected heterozygosity; *H*_O_, average observed heterozygosity; *N*, number of individuals per population used in final analysis (after exclusion of individuals because of low read count or misidentification); *π*, nucleotide diversity.

For **Black Rockfish**, 19,700 loci were retained after filtering, and seven individuals were removed from subsequent analyses due to low read count. Six individuals were outliers based on high scores on PC1 (Figure S8) and had low heterozygosity, positive *F*_IS_ values (Figure S9), and high relatedness (Figure S10). There were also four outliers along PC2, which had heterozygosity and relatedness within the range of the other individuals. None of the outliers had any methodological (sequencing run and well, read depth), genetic (relatedness, outlier loci between groups), or biological peculiarities (size, sampling date) (Figure S11). Nevertheless, we removed both groups of outliers from the dataset. The PCA (Figure 2) suggested one genetic cluster (*K* = 1), using the greatest *L*(*K*), since the Evanno method does not evaluate *K* = 1 (Figure S12); however, pairwise *F*_ST_ estimates showed small (*F*_ST_ = 0.001 for all comparisons) but significant differences between geographic regions (Table 2). We found no chromosomes with highly linked regions (Figure S5), suggesting that there were no chromosomal inversions.

For **Puget Sound Rockfish**, 15,200 loci were retained after filtering. Eight individuals were misidentified as Redstripe Rockfish in the field and were thus removed from analysis (Figure S1). Two individuals were removed due to low read count. We found no evidence of population structure in Puget Sound Rockfish, though they do not occur along the Washington coast. The PCA showed no distinct clustering of individuals and the *STRUCTURE* analysis showed that *K* = 1. All pairwise *F*_ST_ estimates were non‐significant (Table 2). We found no chromosomes with highly linked regions, suggesting that there were no chromosomal inversions.

**FIGURE 3:**
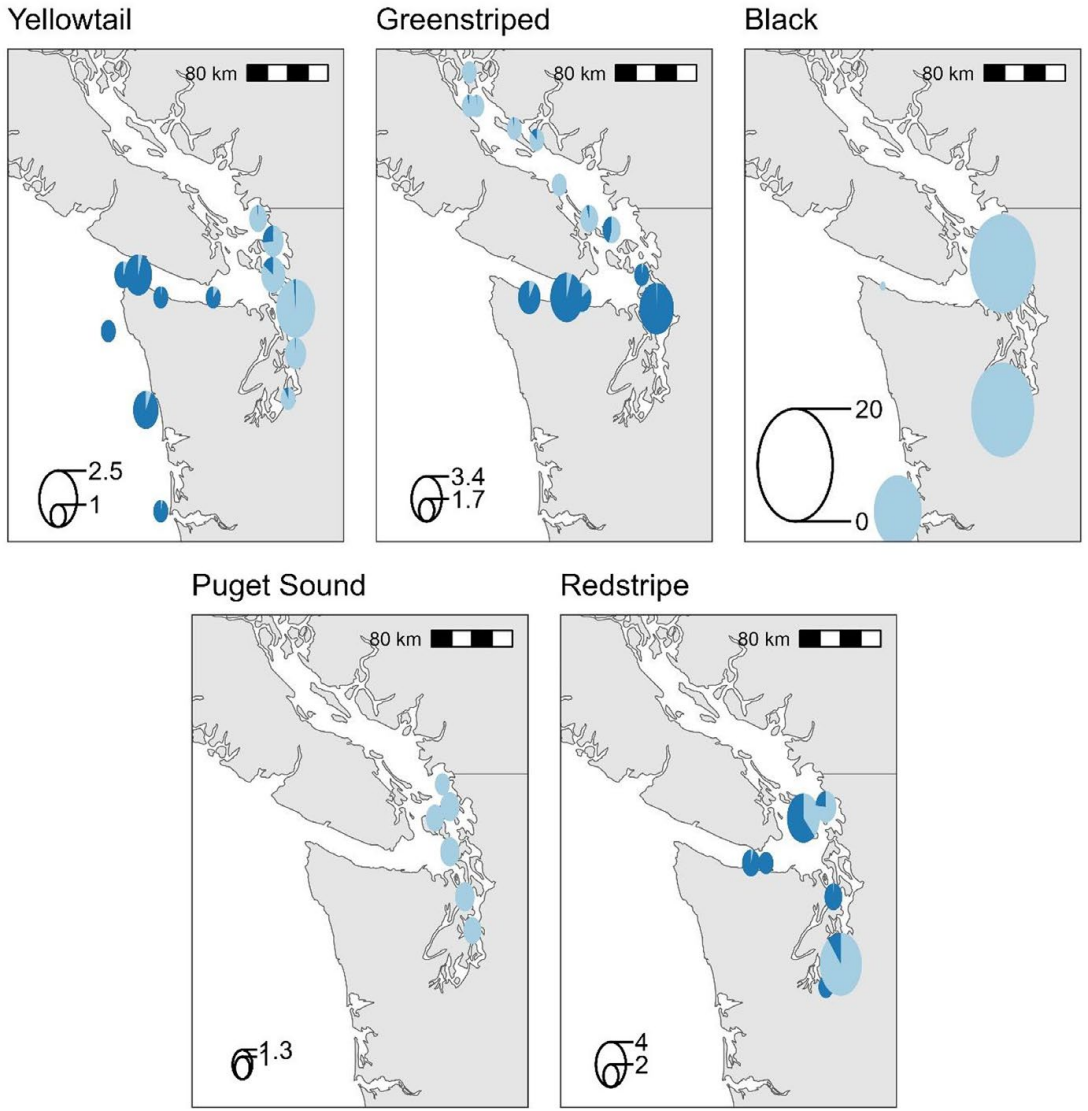
Geographic distribution of STRUCTURE clustering for five species of rockfish. Pie charts are coloured according to *STRUCTURE* plot results (see Figure 2) and their size shows sample size. The pie segments correspond to the average individual admixture proportions in each collection. Similar capture coordinates were pooled into the same pie.

For **Redstripe Rockfish**, 12,275 loci were retained after filtering. Nine individuals were removed from the analysis: six individuals due to low read count and three due to field misidentification. Of the three misidentified individuals, one clustered with Greenstriped Rockfish and two clustered with Puget Sound Rockfish (Figure S1). We found evidence for two genetic clusters in both the PCA and *STRUCTURE* analyses for Redstripe Rockfish (*K* = 2), but there was no clear geographic pattern (Figures 2 and 3). One of the clusters identified in the PCA and *STRUCTURE* analyses was primarily (20/24 individuals, or 83%) sampled in 2014 (Figure S13). Individuals from 2014 were collected in multiple geographic locations and at multiple dates throughout the year and had similar pairwise relatedness to other collection years (Figure S14) suggesting these individuals were not caused by sweepstakes recruitment. However, individual fish were larger and less variable in total length in 2014 (256 mm ± 41) compared to all other years, (231 mm ± 55) suggesting these individuals were of similar age (Figure S15). *N*_*e*_ estimates were 1.7 times larger for the 2014 cluster (1229 ± 4.4) than the other cluster (729 ± 1.7) (Table S2). The *F*_ST_ estimate between the two groups was 0.023 (0.0220–0.0249). We found no chromosomes with highly linked regions (Figure S3), suggesting that there were no large chromosomal inversions. Manhattan plots of *F*_ST_ show that outlier loci are distributed across six chromosomes (Figure S16). Time since divergence calculated from the all‐sites VCF file between the two clusters was estimated at 50,531 years (Table S1).

## 4 Discussion

In this study, we compared the population structure of five common rockfishes of the Salish Sea in relation to life history, physical barriers, and genome structure. Yellowtail and Greenstriped Rockfish showed population structures consistent with known geographic or oceanographic barriers and glacial barriers during the last glaciation. In contrast, Puget Sound and Black Rockfish showed no geographic population structure. Redstripe Rockfish showed temporal genetic structure likely caused by an irregular recruitment event from a population that also predated the last glaciation. There are many possible mechanisms that may act as drivers of population structure in marine species, including extrinsic geographic and environmental barriers (Johansson et al. 2008), but also intrinsic factors such as population history (Canino et al. 2010), life history characteristics such as depth preference (Sivasundar and Palumbi 2010) and the behaviour of larvae and juveniles (Levin et al. 2000), sweepstakes recruitment (Burford and Larson 2007) and chromosomal inversions (Longo et al. 2020). Here we discuss the potential causes for population structure for our study species.

### 4.1 Bathymetry and Oceanography

Oceanographic conditions and bathymetry influence population structure in many marine species, including rockfishes (Andrews et al. 2021; Hess, Vetter, and Moran 2011; Sivasundar and Palumbi 2010). For example, Point Conception in California is known as a biogeographic barrier for Vermilion (*S. miniatus*; Longo et al. 2022), Brown (Buonaccorsi et al. 2005), and Grass Rockfish (*S. rastrelliger*; Buonaccorsi et al. 2003). Similar biogeographic boundaries are known in the Salish Sea, a unique glacier‐carved inlet that is comprised of several discrete basins separated by shallow sills with only one large connection to the ocean (Moore et al. 2008). These bathymetric conditions create circulation patterns that limit planktonic dispersal and thus create strong zoogeographic breaks (Engie and Klinger 2007). As a result, many marine species may have distinct coastal and Puget Sound populations, including Pacific Cod (Drinan et al. 2018), Yelloweye Rockfish (Andrews et al. 2018), Dungeness Crab (Jackson and O'Malley 2017) and Pacific Hake (Iwamoto, Ford, and Gustafson 2004). Genetic differentiation in Yellowtail and Greenstriped Rockfish followed this pattern, suggesting that similar geographic and oceanographic conditions may determine population structure in these species.

In Greenstriped Rockfish, our results suggested a barrier in the San Juan Islands between the Strait of Georgia Basin (British Columbia) and the San Juan Island Basin (USA), which may be caused by restricted larval dispersal. A similar barrier was predicted from oceanographic models in Yelloweye Rockfish (Andrews et al. 2021) due to the circulation in the area, which restricts nearly half of the bottom layer inflow from the Juan de Fuca Strait and south/central Puget Sound (Khangaonkar, Long, and Xu 2017). These conditions result in a high residence time (160 days) within the Strait of Georgia, which is 70 days higher than any other basin in Puget Sound (Pawlowicz, Hannah, and Rosenberger 2019). In addition, there is only one deep water passage between the deeper British Columbia waters and the Strait of Juan de Fuca (Khangaonkar, Long, and Xu 2017). Shallow regions within the San Juan Islands could restrict adult movement of deep‐water species, such as the Greenstriped Rockfish (Love, Yoklavich, and Thorsteinson 2002). As Greenstriped Rockfish was the only species collected from British Columbia, other species may also be affected by that barrier and international collaboration will be required to properly assess the genetic structure of rockfishes throughout the Salish Sea.

The only species in our study that showed patterns of genetic structure aligning with previous assumption of Salish Sea DPSs was Yellowtail Rockfish, with an apparent barrier across the Victoria Sill, a shallow sill (~55 m, Ryan et al. 2019) that separates the Salish Sea from the Strait of Juan de Fuca and the Washington Coast. The Victoria Sill causes considerable vertical mixing in the Strait of Juan De Fuca (Khangaonkar, Long, and Xu 2017), influencing salinity (Masson and Cummins 2000), primary productivity (Masson and Peña 2009), and larval retention times (Engie and Klinger 2007). Thus, the combination of shallow water and a shift in current patterns could function as a barrier limiting both adult and larval dispersal. Indeed, Yelloweye Rockfish (Andrews et al. 2018) and Pacific Cod (Drinan et al. 2018) show genetic differentiation across this sill as well.

Despite the ubiquity of geographic barriers to gene flow and dispersal, as well as the apparent similarity in life history characters, our results suggest species‐specific genetic breaks. Similar species‐specific barriers have been identified in the Baltic Sea (Wennerström et al. 2013), as well as along large coastlines for both rockfishes (Longo et al. 2022; Sivasundar and Palumbi 2010) and intertidal invertebrates (Kelly and Palumbi 2010). Many marine barriers are permeable/soft barriers, and as such their effectiveness depends on a species' life history characteristics such as larval behavior, settlement behavior (Sivasundar and Palumbi 2010), parturition timing (Shanks and Eckert 2005), and adult movement (Delaval et al. 2018). Our results suggest that relatively small differences between species in these life history characteristics can cause considerable differences in population structure.

### 4.2 Life History Characteristics

Explicit and quantitative analyses to compare differences in life history characteristics require large data sets that evaluate correlations between many species and traits. For example, one study using 74 marine finfish correlated various life history characteristics and biological traits with population genetic structure (Cusa et al. 2022). Due to the limited number of species, we could not make such quantitative comparisons. Nevertheless, we provide the following qualitative comparisons based on known life history characteristics summarised in Table 3.

**TABLE 3:** Summary of life history characteristics of eight Puget Sound rockfishes.

Species	Depth^1^	Population size^2^	Adult habitat^1^	Site fidelity^3,4^	Peak parturition^1^	Larval duration^5^	Population structure
Black	S	P	D	M	I‐V	L	N
Puget Sound	S	P/H	P	—	VIII‐IX	—	N
Canary	D	D	P	L	I‐II	L	N
Yellowtail	D	P	D	—	I‐IV	L	Y
Greenstriped	D	H	B	M	VI	S	Y
Redstripe	D	H	D	—	IV‐VII	L	Y
Yelloweye	D	D	B/D	H	V‐VI	L	Y
Quillback	M	P/V	B	H	IV	L	Y

*Note:* Depth (Shallow (S): < 50 m, midwater (M): 50–100 m, deep (D): 100+ m); population size (healthy (H), precautionary (P), vulnerable (V) and depleted (D)); adult habitat (benthic (B), demersal (D), pelagic (P)); site fidelity (high (H): > 25 m, moderate (M): 15–25 m, low (L): > 15 m); peak parturition (calendar month); larval duration (long (L): > 2 months, short (S): ≤ 2 months). Depth, adult habitat, and peak parturition are from Love, Yoklavich, and Thorsteinson (2002)^[1]^. Population size estimates are from Palsson et al. (2009)^[2]^. Site fidelity estimates are from Hannah and Rankin (2011)^[3]^ and Lowe et al. (2009)^[4]^. Larval duration estimates are from Carr and Syms (2006)^[5]^. The presence of population structure is based on results from Figure 2, Table 1, Wray et al. (2024), and Andrews et al. (2018). Copper and Brown Rockfish are not included in the table due to the influence of hybridisation on population structure (see Wray et al. 2024).

Adult depth and habitat preference are relatively well known and may explain higher gene flow in Black Rockfish when compared to Yellowtail Rockfish (Hess, Vetter, and Moran 2011; Hess, Hyde, and Moran 2023) along the US West Coast. In our study, the shallow water and/or pelagic species (Black and Puget Sound) lacked genetic differentiation, while deep‐water benthic or demersal species showed some degree of genetic structure (Redstripe, Greenstriped, Yellowtail) (Table 3). However, geographic patterns of genetic structure differed between the deep‐water species, suggesting adult depth is not the only driving factor for population structure. Furthermore, the deep‐water Canary Rockfish do not show any genetic population structure (Andrews et al. 2018). This pattern continues with adult habitat preference, since both pelagic species (Canary and Black) show no population structure but our benthic species have varying levels of population structure. This ambiguity of depth preference on population structure is also evident in Sivasundar and Palumbi (2010) for 15 species of rockfish along the coast of Oregon and California. However, the effect of bathymetry on genetic population structure may be vastly different along the open coast and the estuarine environment such as Puget Sound. The existence of population structure in fjords of deeper water species has been reported in other marine species such as Copper Rockfish (Dick, Shurin, and Taylor 2014), Pacific hake (García‐De León et al. 2018), and Atlantic (*Gadus morhua*; Jorde et al. 2007) and Pacific cod (Cunningham et al. 2009). As this research is expanded to include other fish in Puget Sound, including other rockfish species, additional insights may be gained regarding the influence of fjord‐like systems on the population structure of deep‐water species.

Timing of larval release is not well known in most rockfishes, and may vary regionally and locally (Love, Yoklavich, and Thorsteinson 2002), but may be very important in determining interspecific differences in genetic structure (Doherty, Planes, and Mather 1995). Timing of parturition has been shown to be an important factor in the Salish Sea (Andrews et al. 2021), where there are drastic seasonal differences in oceanographic properties including salinity, temperature, dissolved oxygen and water density (Moore et al. 2008). Off the coast of California, USA, such seasonal differences in oceanographic conditions have been shown to impact gene flow between populations of a single fish species (Jackson, Roegner, and O'Malley 2018) and explain differences in population connectivity between multiple species of fish with similar life history characteristics (Shanks and Eckert 2005). In our study, species that released larvae in spring and summer were more likely to show population structure than those with peak parturition in winter (Table 3).

Larval duration is the most obvious predictor of population structure (Selkoe and Toonen 2011), but is poorly known in the genus *Sebastes*, relatively similar among species, and may vary latitudinally and regionally within species. Furthermore, larvae and pelagic juveniles of some rockfishes are relatively strong swimmers that may not drift like passive particles but are able to swim against currents (Kashef et al. 2014) and certainly can change depth to influence dispersal (Leis 2006). Correspondingly, we did not find any patterns suggesting that pelagic larval duration can predict the existence of population structure in *Sebastes* spp., though there was only one species with confirmed short larval duration (Table 3).

### 4.3 Population History

Current population structure and genetic diversity depend not only on current connectivity but also on historical demographic events and climate patterns (Hauser and Carvalho 2008). In particular, the impact of past ice ages on genetic population structure is highly species‐specific and likely influenced by a species' life history characteristics and physiological tolerances (Bernatchez and Wilson 1998). In the North Pacific, the Pleistocene ice ages glaciated most of Canada and parts of northern Washington (Porter 1977), but populations may have persisted in isolated glacial refugia (Shafer et al. 2010). In some instances, refugial populations are now in panmixia (such as in the catadromous European eel [Dannewitz et al. 2005]), likely due to high gene flow in the marine environment. In other species, recolonisation from different refugia in combination with some barriers to gene flow may result in a mosaic of genetically differentiated populations. In our study, we found that Redstripe and Greenstriped divergence time estimates pre‐date the most recent glacial expansion which covered much of Puget Sound and the Strait of Juan De Fuca (Mann and Gaglioti 2024). In contrast, the two populations of Yellowtail Rockfish diverged more recently, corresponding with the advancement of the Puget Sound glacial lobe approximately 16.6–20.9 kya (Mann and Gaglioti 2024), which may have separated populations into different glacial refugia. It therefore seems likely that the genetic structure in all three species may have been a consequence of separation in different glacial refugia and subsequent secondary contact. This interpretation leaves the interesting hypothesis that population structure may also exist in other species (e.g., Black Rockfish) but is not detectable by RADseq because of recent post‐glacial separation of these populations. More involved analyses such as genetic parentage or kin structure (Hess 2010; Baetscher et al. 2019) could address this question. We did not identify any pattern between the estimated time of divergence and life history characteristics (Table 3).

Populations originating from separate glacial refugia could represent a significant evolutionary legacy of the species (Serrao, Reid, and Wilson 2018), which could provide increased adaptive potential. This increase in adaptive potential is significant for the recovery of a population and for the ‘significance’ criterion needed to list populations as a DPS under the ESA (Fay and Nammack 1996) and is thus highly relevant for conservation.

### 4.4 Temporal Genetic Variation

Small‐scale genetic differentiation in populations with presumed high gene flow, or chaotic genetic patchiness (Johnson and Black 1982), has been frequently observed in marine fish species (Burford Reiskind, Carr, and Bernardi 2011; Gilbert‐Horvath, Larson, and Garza 2006; Larson and Julian 1999; Selwyn et al. 2016). In Redstripe Rockfish, patterns of chaotic genetic patchiness were largely explained by the likely presence of genetically differentiated cohorts. In particular, individuals collected in 2014 were genetically distinct from all other year classes. Those individuals were primarily between 25 and 30 cm in length which is close to the size at first sexual maturity (Love, Yoklavich, and Thorsteinson 2002). Additionally, the variance in body size in the 2014 individuals was 2.5 times smaller than in the mixed‐year cluster, suggesting that the 2014 individuals were all from the same year class. Other *Sebastes* species show multiple genetically different larval clusters in a homogeneous adult population in northern California, specifically for juveniles of the 2000 year class (Burford and Larson 2007; Burford Reiskind, Carr, and Bernardi 2011). Furthermore, Redstripe Rockfish along the western coast of Vancouver Island showed high variation in year class strength, with documented surges of recruits in 2000 and 2007 (Star and Haigh 2021).

There are four potential explanations for this small‐scale genetic variation in large homogeneous marine populations: (1) temporal variation in currents introducing foreign genotypes to new areas, (2) natural selection acting on larvae prior to settlement, (3) variation in reproductive success among adults (Larson and Julian 1999) or (4) the presence of a cryptic species. The introduction of foreign genotypes hypothesis would assume there is an undetected population of genetically divergent Redstripe Rockfish that periodically enters the Puget Sound region. Such a genetically divergent population may occur either along the coast north or south of the Strait of Juan de Fuca or in the British Columbia waters of the Salish Sea. Natural selection in the larval stage would cause differentiation at few outlier loci rather than across the genome (Lewontin and Krakauer 1973). Our study supported the effect of selection by revealing multiple outlier loci across six chromosomes (Figure S16). However, more research is necessary to conclusively demonstrate selection. Third, variation in reproductive success, commonly referred to as sweepstakes recruitment (Hedgecock 1994), would cause very low effective population size estimates since very few parents successfully produced offspring. In contrast, our results suggest that the 2014 individuals come from a large parental population (Table S2), reducing the possibility of sweepstakes recruitment. Finally, an alternative explanation for the temporal differentiation in Redstripe Rockfish could be the presence of a cryptic species, similar to that described in Blue Rockfish (*Sebastes mystinus*) (Burford Reiskind, Carr, and Bernardi 2011). Indeed, *F*_ST_ values between the two clusters (Figure S16) are an order of magnitude higher than any other pairwise *F*_ST_ values (Table 2). On the other hand, they were lower than commonly reported between species and even between geographically overlapping populations within species (Longo et al. 2022). Additionally our interspecific PCA analyses showed no evidence of cryptic species (Figure S1), and the divergence time between the two genetic clusters is similar to that of the Greenstriped Rockfish populations (Table S1). As such, we hypothesise this temporal variation is due to a wave of immigration from a single class introducing novel genotypes. The lack of gene flow between individuals collected in 2014 and other year classes can be explained by the age of the 2014 fish, which at the time of sampling likely had just reached maturity and their offspring were too small to be sampled by hook and line.

Similar temporal processes cannot be excluded for other rockfish species. For example, all Greenstriped Rockfish from British Columbia were caught in the same year and were of similar size (Figure S13). Similarly, all of the Washington Coast samples in Yellowtail Rockfish were caught in the same year, but they have a wide range of sizes suggesting that they originate from different recruitment events (Figure S17). It is therefore possible that the proposed geographic structure in Greenstriped and Yellowtail rockfish is also due to year class patterns, similar to Redstripe Rockfish. Due to the presence of well‐established biogeographic barriers to dispersal in this region, however, the genetic differentiation more likely represents spatial rather than temporal structure. Nevertheless, the distinction of temporal and spatial patterns is a well‐established complication in population genetic studies of marine species (Waples 1998), and additional studies to investigate temporal genetic variation further are needed.

### 4.5 Barriers to Gene Flow Despite Dispersal

Despite the sharp genetic boundaries in population structure in Yellowtail and Greenstriped Rockfish, we found evidence from both PCA and *STRUCTURE* for dispersal between populations in both species. In particular, three Yellowtail Rockfish with West Coast ancestry were found in the Salish Sea (9% of all individuals) while four West Coast individuals showed partial Salish Sea ancestry (20% of individuals, Figure 2). One Greenstriped Rockfish from British Columbia had Puget Sound or West Coast ancestry (10%, Figure 2). Similarly, dispersers from the Salish Sea to the West Coast (9%) were detected in Yelloweye Rockfish (Andrews et al. 2018). Such high immigration rates are expected to erode any remnant genetic differentiation between glacial refugial populations relatively quickly, yet these populations are still very differentiated. Multiple intrinsic and extrinsic reproductive barriers may limit interbreeding between populations and thus explain such dispersal without or with very little gene flow. Phenotype‐environment mismatches may significantly reduce gene flow by selecting against immigrants and hybrids (Marshall et al. 2010). For example, environmental barriers to connectivity were found for diverse marine species such as Atlantic herring (*Clupea harengus*; Limborg et al. 2012), Pacific cod (Drinan et al. 2018; Fisher et al. 2022), and lobster (*Homarus americanus*; Benestan et al. 2016). Selection was also implicated in the isolation between Atlantic *Sebastes* ecotypes (Benestan et al. 2021). Especially in environments as different as the open coast and freshwater‐influenced estuaries, such selective constraints may cause a significant reduction in connectivity (Limborg et al. 2012; Berg et al. 2015). In fact, the temporal differentiation among our results from Redstripe Rockfish shows clear signs of selection (Figure S15). This may suggest that selection may play a role in the population structure seen, as we identified multiple clusters of SNPs with high *F*_ST_. In addition, rockfish have complex mating rituals (Helvey 1982) and apparent mate choice (Johansson et al. 2012), so it is possible that separate mating rituals in the two populations could influence mate choice and reinforce population differentiation. In any case, the indication of reproductive barriers suggests that the population grouping detected here could be categorised as ‘distinct’ and therefore qualify as a DPS under the ESA.

Genetic connectivity and gene flow may also depend on intrinsic features of the genome, such as chromosomal inversions that can induce hybrid sterility and facilitate the maintenance of co‐adapted gene complexes (Faria and Navarro 2010) and promote local adaptation (Wellenreuther and Bernatchez 2018). By suppressing recombination, chromosome inversions create supergenes, clusters of genes that are inherited as a single unit and thus may facilitate adaptation and divergence at multiple traits even in the presence of gene flow (Jay et al. 2018). Chromosomal inversions contribute to population structure in Pacific Herring (*Clupea pallasii*; Petrou et al. 2021), Capelin (*Mallotus villosus*; Cayuela et al. 2020), and Lingcod (Longo et al. 2020) where they control migratory behaviour, low salinity tolerance, and spawn timing. However, we did not detect any large chromosomal inversions in the five rockfish species studied (Figure S4). Although whole genome sequencing may reveal smaller chromosomal inversions not detectable by RADseq (Andrews and Luikart 2014), it stands to argue that only large inversions link an adequate number of genes to affect local adaptation. The absence of large inversions in the genome of the rapidly speciating *Sebastes* genus demonstrates the potential of genetic population structure and adaptive radiation in high gene flow species even without large chromosomal inversions.

Our inversion analysis did however reveal a sex‐linked region of Chromosome 16 in Greenstriped Rockfish. Some rockfishes have a nascent Y chromosome sex determination system, evidenced by highly linked chromosome segments with low heterozygosity in females and high heterozygosity in males (Fowler and Buonaccorsi 2016). Sex‐determining regions in Rockfish species are highly variable in their location and effect (Sykes et al. 2023), which may explain why this region was not found in our other species.

### 4.6 Implications for Fisheries Management

Species‐specific patterns of population structure pose special challenges for fisheries management by requiring independent strategies. The *Sebastes* species complex in Puget Sound exemplifies this challenge. Of the species studied here, only Yellowtail Rockfish corresponded to the current federal assumptions of rockfish DPS boundaries, which were based on three species known to hybridise within Puget Sound (Brown, Copper, and Quillback Rockfish) (Buonaccorsi et al. 2002, 2005; Seeb 1998). These assumptions were also instrumental in the ESA listing decisions for Yelloweye, Canary, and Boccacio Rockfish (Drake et al. 2010) even though there was no empirical evidence supporting these DPS boundaries. The boundaries were confirmed for Yelloweye Rockfish, but not for Canary Rockfish (Andrews et al. 2018) which was subsequently delisted. Therefore, out of the 10 species for which genetic data were accumulated after the designation in 2010, eight showed no or different genetic differentiation. Our study suggests that population structure inference cannot be made between species, even if they are closely related, have similar life history, and are occupying the same environment.

Patterns of population structure differed widely among *Sebastes* species. Such species‐specific geographic barriers pose a special challenge for spatial conservation measures such as marine protected areas (MPAs). For example, if multiple species show similar patterns of population connectivity, a single MPA could be established to protect all species (Abecasis, Afonso, and Erzini 2014). If, on the other hand, population structure differs among species and the processes of differentiation are different, multiple MPAs may be needed to conserve the maximum DPS diversity for all species. As such, multiple MPAs across Puget Sound and the Washington Coast may be necessary for the conservation of all rockfish species.

Recovery of Puget Sound rockfishes and the potential re‐establishment of a recreational fishery may depend on recruitment subsidies from healthy Washington Coast populations. Genetic data can provide valuable cues on demographic connectivity, but the relationship between ecological and genetic populations is complex and depends on population sizes, population history, and the relative fitness of dispersers. Nevertheless, our results suggest that the likelihood of such recruitment subsidies differs between species. For Black Rockfish, no genetic differentiation was found between coastal and Puget Sound populations, which may indicate demographic connectivity between these two areas. As historical factors and large population size may mask subtle population structure (Hauser and Carvalho 2008), such an assertion would have to be confirmed with tagging or dispersal studies. In contrast, we detected only three potential Washington coast dispersers out of 23 in the Puget Sound population of Yellowtail Rockfish including one sample with mixed coastal and sound ancestry. Although this translates to 13% of the population, our sample sizes are likely too small to infer dispersal rates across multiple years. Nevertheless, such coastal immigrants could support a fishery within Puget Sound (especially if they are not reproductively successful and thus do not contribute to recruitment), but additional studies are needed to inform a formal stock assessment. Finally, our study identified the potential for large sporadic subsidies from genetically distinct populations of Redstripe Rockfish (and possibly Greenstriped Rockfish). Such sporadic and unpredictable recruitment pulses pose their own challenges for stock assessments, which should be addressed in conjunction with genetic identification of immigrants.

Species‐specific management of rockfishes is complicated by the high rate of misidentification of rockfishes. Approximately 8% of our individuals were misidentified, even though they were collected by professional samplers, who have a significantly lower rate of misidentification compared to recreational fishers (Beaudreau, Levin, and Norman 2011). Both management and conservation of species rely heavily on accurate species identifications, and the species‐specific population structure in Puget Sound rockfishes revealed in our study emphasises the need for accurate species identifications to properly assess and manage them. The genetic markers developed here provide a reliable method to identify species, populations, and dispersers and thus may prove to be a useful tool for future research, ultimately leading to more informed and effective strategies to facilitate the recovery of rockfish populations in Puget Sound. Rapid advances in DNA technology may soon provide hand‐held devices that allow in‐field genetic identification at a reasonable cost (Baerwald et al. 2020).

## Author Contributions

The authors confirm contribution to the paper as follows: study conception and design: A.W., L.H.; sample collection: A.W., R.P., L.L., K.M.N., D.H.; data collection: A.W.; analysis and interpretation of results: A.W., L.H., E.P.; draft manuscript preparation: A.W. All authors reviewed the results and approved the final version of the manuscript.

## Conflicts of Interest

The authors declare no conflicts of interest.

## Benefits Statement

This work demonstrates a collaboration between state, federal, and international organisations who have direct impacts on the management of this region and these species. The results of this study have been provided to relevant stakeholders and will be utilised if a stock assessment of these species is deemed appropriate. Finally, as described above, the methods and relevant data files will be made publicly available (upon acceptance).

## Supporting information


Data S1

## Data Availability

All genetic data (pre‐ and post‐filtering) and relevant metadata are available on Dryad (DOI: 10.5061/dryad.866t1g1xj). All scripts used in this manuscript are available on GitHub (https://github.com/anita‐wray/rockfish_RADseq). This repository contains the raw fastq files (gzipped), end product files (in VCF format) and relevant metadata on the final samples used in the results. Raw fastq files are also available on the NCBI SRA Accession number: PRJNA1145982 (Wray 2024a, 2024b).
