# Supplementary material for: Divergent Population Structure in Five Common Rockfish Species of Puget Sound, WA Suggests the Need for Species‐Specific Management
Source: Mol Ecol. 2024 Nov 25;34(1):e17590. doi: 10.1111/mec.17590 (PMC11665494; doi:10.1111/mec.17590)
Supplement: Supplementary file 1 — Data S1 [file MEC-34-e17590-s001.docx]

**Supplemental Information for:**

**Divergent Population Structure In Five Common Rockfish Species Of Puget Sound, WA Suggests The Need For Species-Specific Management**

Anita Wray, Eleni Petrou, Krista M. Nichols, Robert Pacunski, Larry LeClair, Kelly S. Andrews, Dana Haggerty, Lorenz Hauser

**Table of Contents:**

| Supplemental Figure 1 Principal components analysis of eight species of rockfish suggests misidentified individuals | Page 2 |
| --- | --- |
| Supplemental Figure 2 *F*_st_ Manhattan Plot of Yellowtail Rockfish clusters | Page 3 |
| Supplemental Figure 3 Principal components analysis of Greenstriped Rockfish labeled by life history characteristics | Page 4 |
| Supplemental Figure 4 Distribution of fish lengths for BC and non-BC Greenstriped Rockfish | Page 5 |
| Supplemental Figure 5 Linkage decay plots per chromosome for five species of rockfish | Page 6 |
| Supplemental Figure 6 Principal components analysis and linkage heatmap of Chromosome 16 in Greenstriped Rockfish. | Page 7 |
| Supplemental Figure 7 Distribution of individual heterozygosity at highly linked region of Greenstriped Rockfish Chr16 | Page 8 |
| Supplemental Figure 8 Principal components analysis of Black Rockfish including outlier individuals with very high homozygosity | Page 9 |
| Supplemental Figure 9 *F*_IS_ in Black Rockfish in relation to read depth | Page 10 |
| Supplemental Figure 10 Pairwise relatedness estimates Black Rockfish individuals and outliers | Page 11 |
| Supplemental Figure 11 Principal components analysis of Black Rockfish labeled by life history characteristics | Page 12 |
| Supplemental Figure 12 Mean likelihood and ΔK for STRUCTURE replicate runs for Yellowtail, Greenstriped, Black, Puget Sound and Redstripe Rockfish | Page 13 |
| Supplemental Figure 13 Principal components analysis of Redstripe Rockfish labeled by life history characteristics | Page 14 |
| Supplemental Figure 14 Pairwise relatedness estimates for 2014 Redstripe Rockfish individuals | Page 15 |
| Supplemental Figure 15 Distribution of fish lengths for 2014 Redstripe Rockfish individuals compared to other sampling years | Page 16 |
| Supplemental Figure 16 *F*_st_ Manhattan Plot of Redstripe Rockfish clusters | Page 17 |
| Supplemental Figure 17 Principal component analysis of Yellowtail Rockfish labeled by life history characteristics | Page 18 |
| Supplemental Table 1 d_xy_ and divergence time estimation based on clustering pattern | Page 19 |
| Supplemental Table 2 N_e_ estimation based on clustering pattern | Page 19 |

**
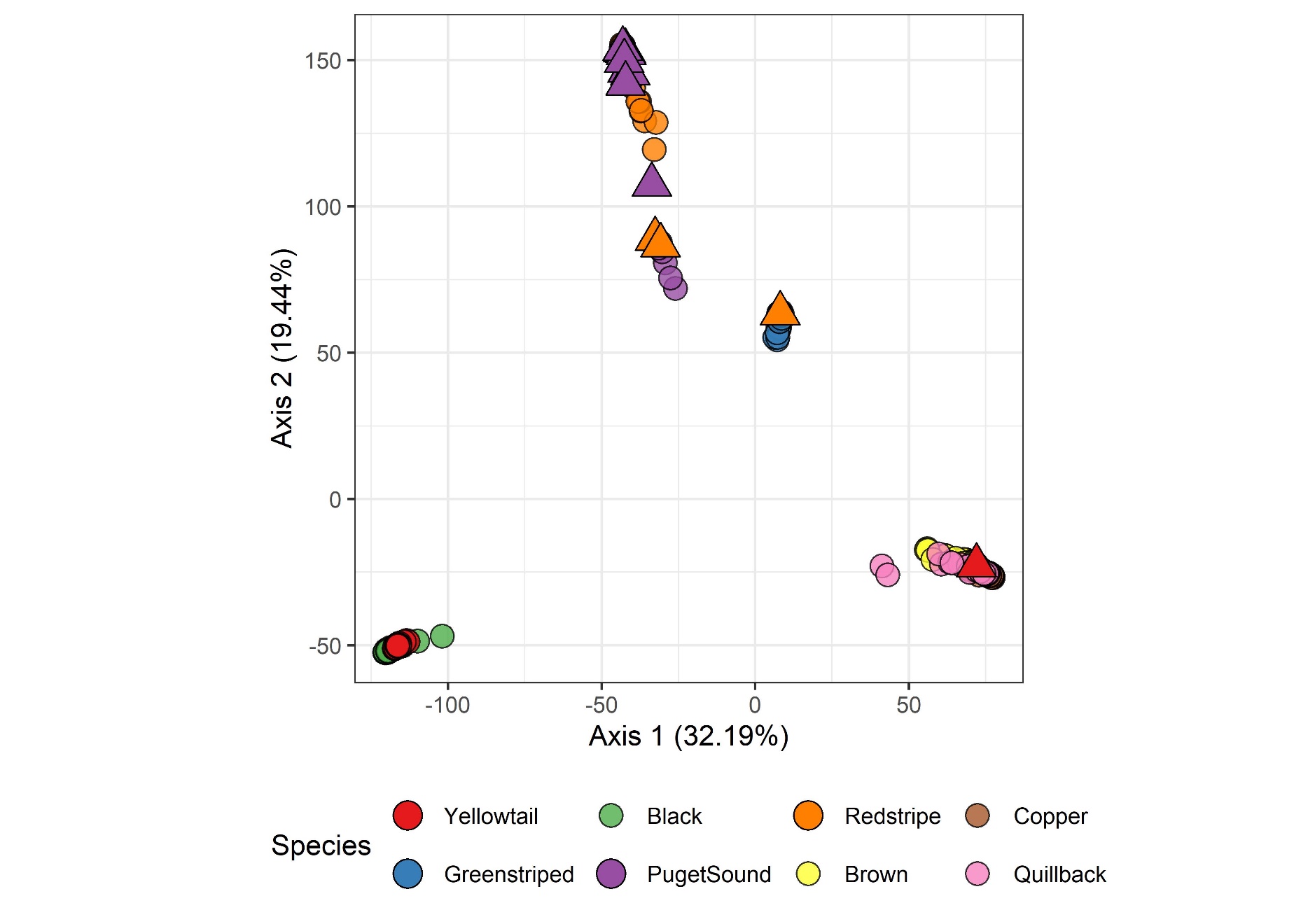
**

**Supplemental Figure 1 Principal components analysis of eight species of rockfish suggests misidentified individuals.** Each circle represents an individual fish, colored according to field species identification. Each triangle represents an individual where field and genetic identification disagreed. The misidentified Yellowtail Rockfish clustered with Brown Rockfish along subsequent PC axes (data not shown).

**
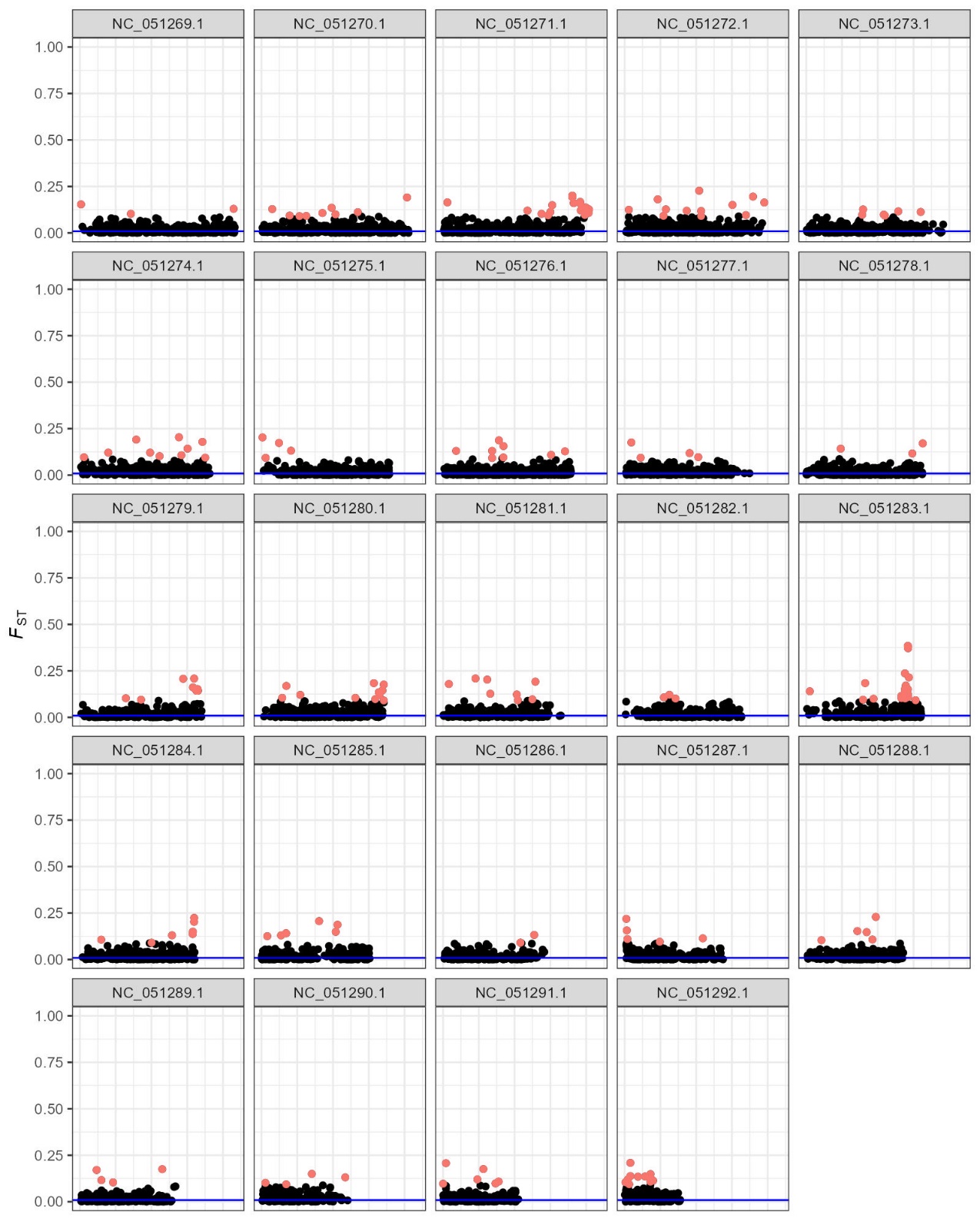
**

**Supplemental Figure 2 Manhattan *F*_ST_ Plot of two geographically divergent groups in Yellowtail Rockfish.** *F*_ST_ was calculated using *hierfstat*. Overall *F*_ST_ between the two groups was 0.009 (0.0079-0.0094, range after 1,000 bootstrap iterations) and is displayed as the blue horizontal line. Points are highlighted in red if they were 10x greater than the overall average *F*_ST_ or 0.09.


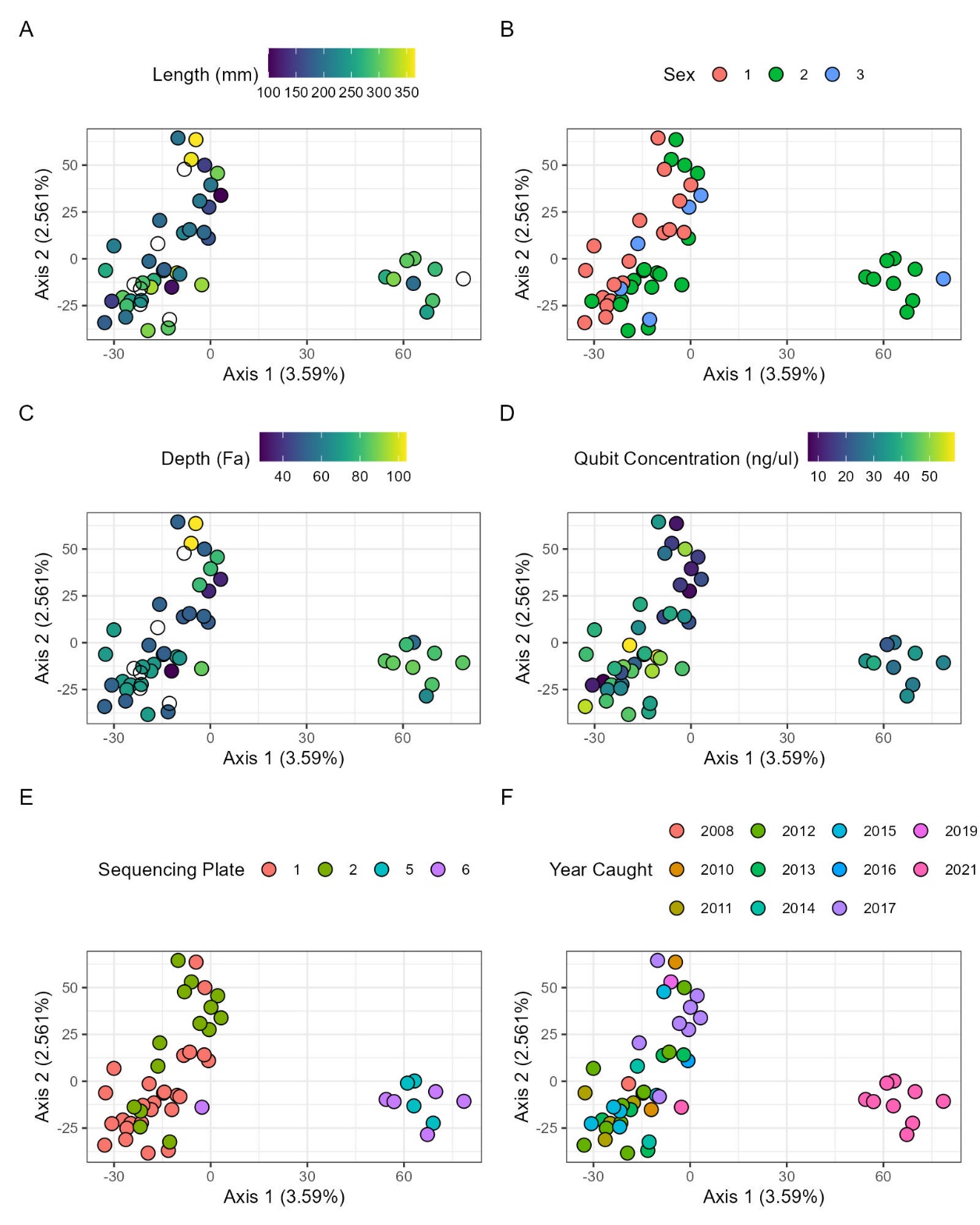


**Supplemental Figure 3 Principal components analysis of Greenstriped Rockfish labeled by life history characteristics.** Each point represents an individual fish, colored by various life history and methodology characteristics. Unfilled circles show missing data. A: length of fish (millimeters), B: sex (1 = Female, 2 = Male, 3 = Unknown), C: depth caught (fathoms), D: extraction Qubit concentration (nanograms per microliter), E: sequencing plate, F: year caught.


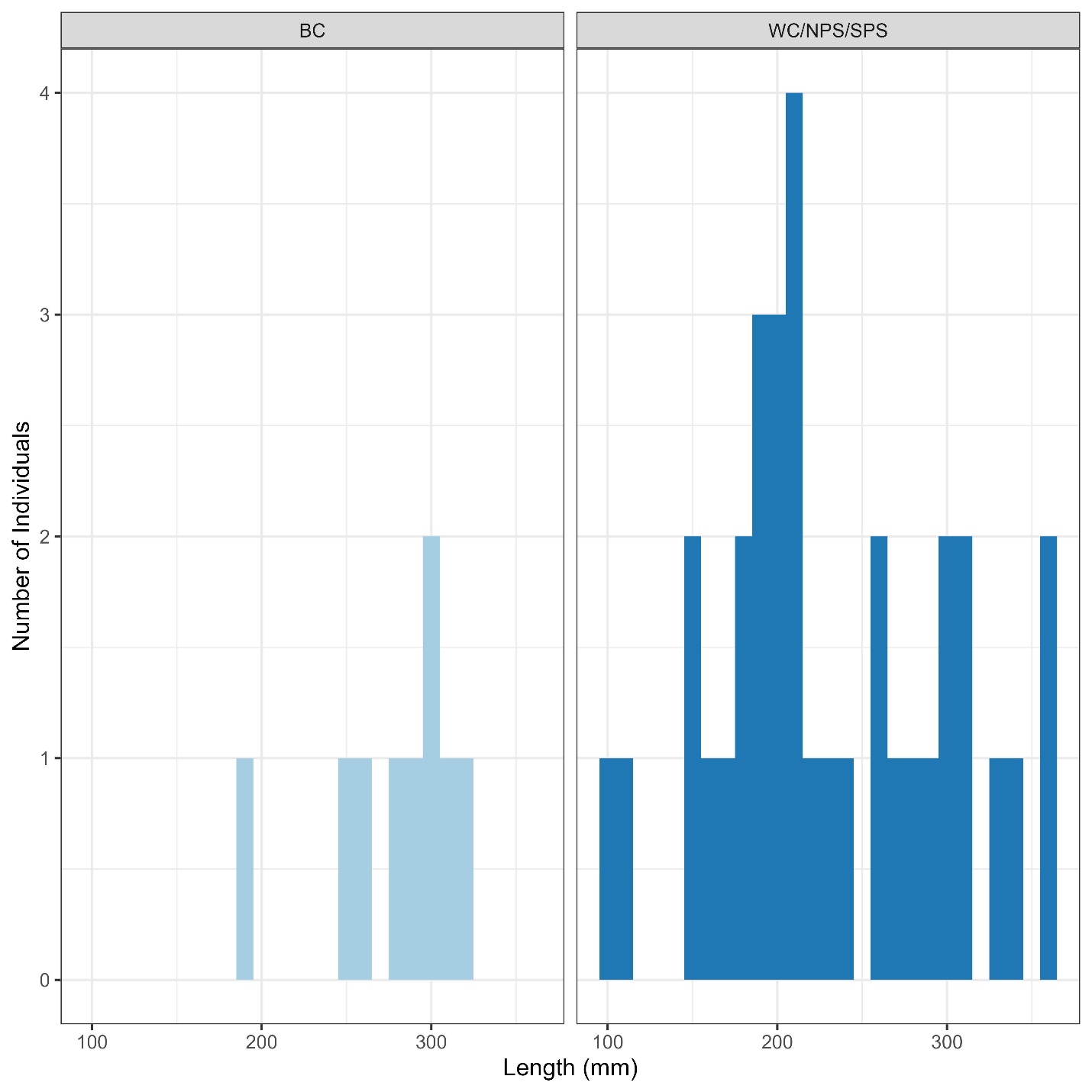


**Supplemental Figure 4 Distribution of fish lengths for BC and non-BC Greenstriped Rockfish.** An ANOVA test revealed no significant difference between lengths of the two groups (F = 3.845, *p =* 0.0567).

**Supplemental Figure 5 Linkage decay plots per chromosome for five species of rockfish.** R^2^ values were calculated using *PLINK*. One chromosome with a block of highly linked loci was identified on Chromosome 16 for Greenstriped Rockfish.


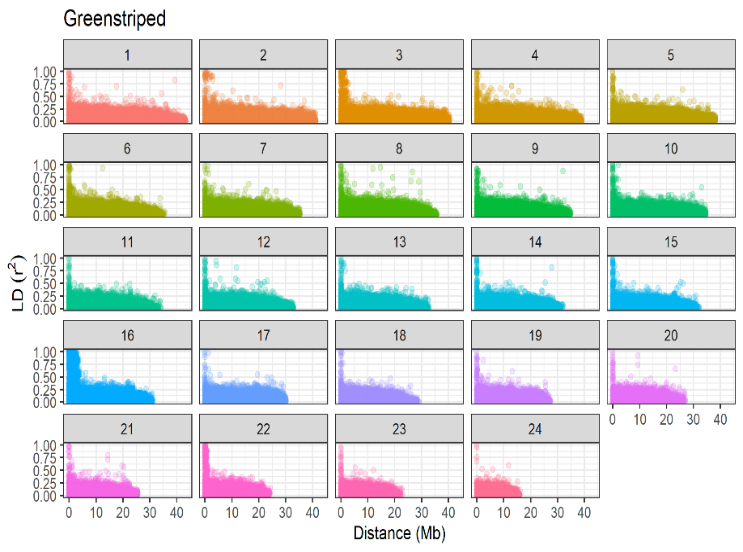

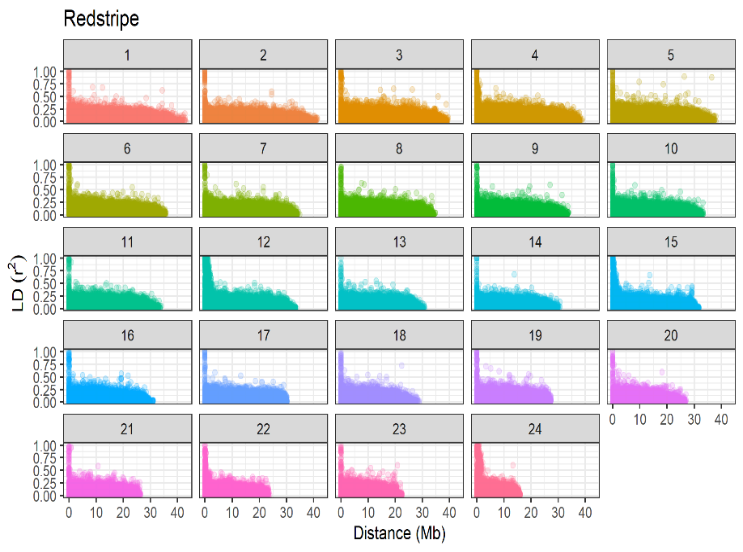

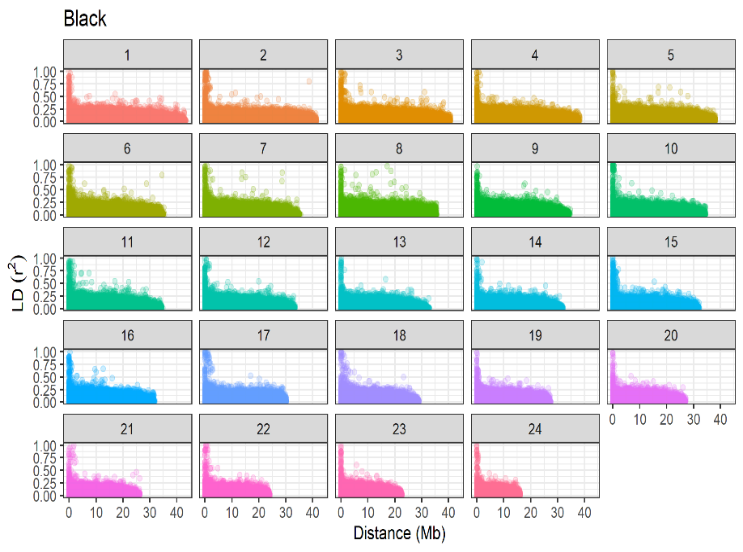

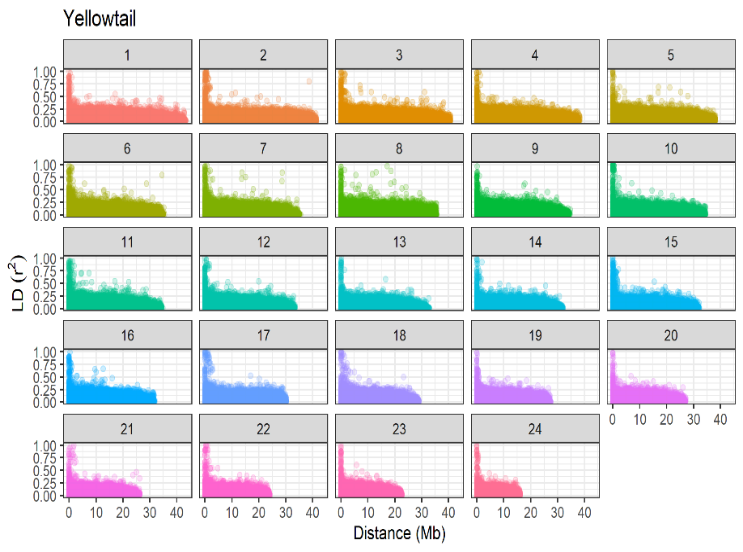

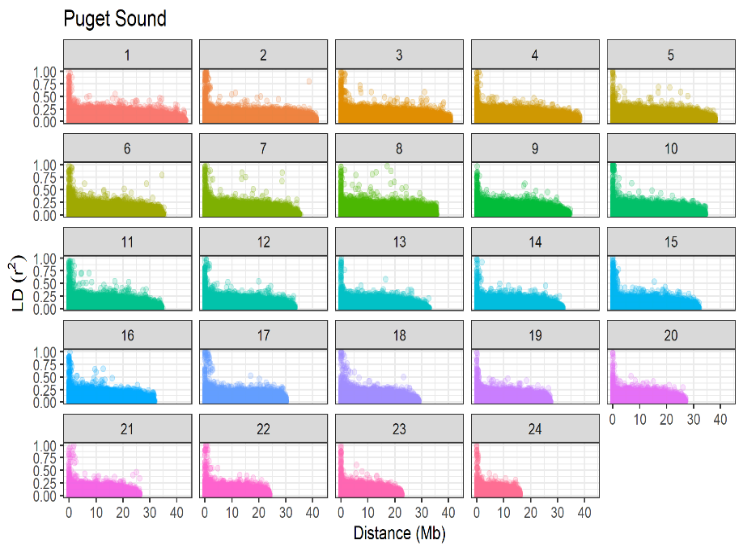


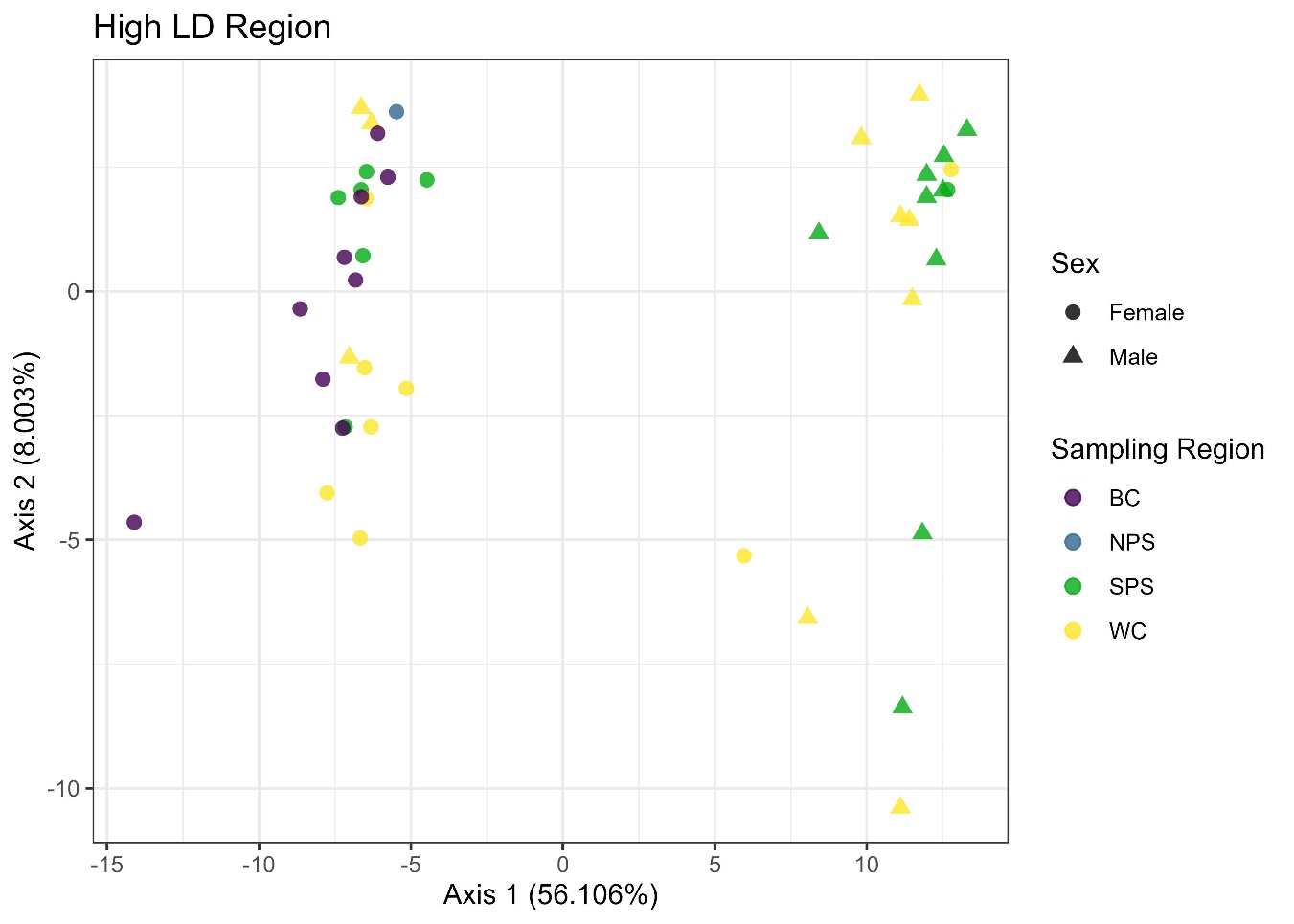

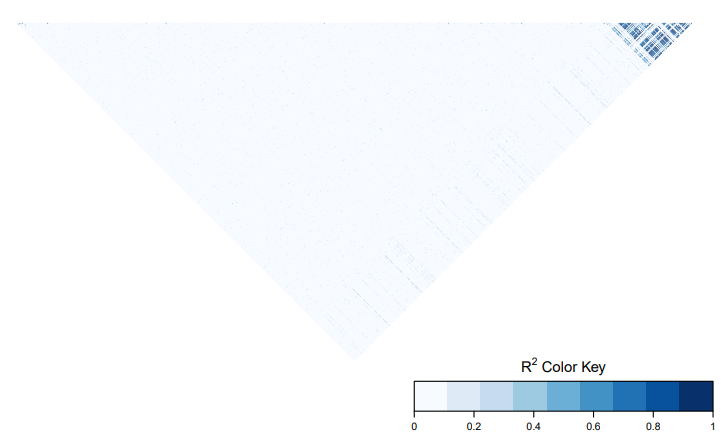


**Supplemental Figure 6 Principal components analysis (top) and linkage heatmap (bottom) of Chromosome 16 in Greenstriped Rockfish.**

**
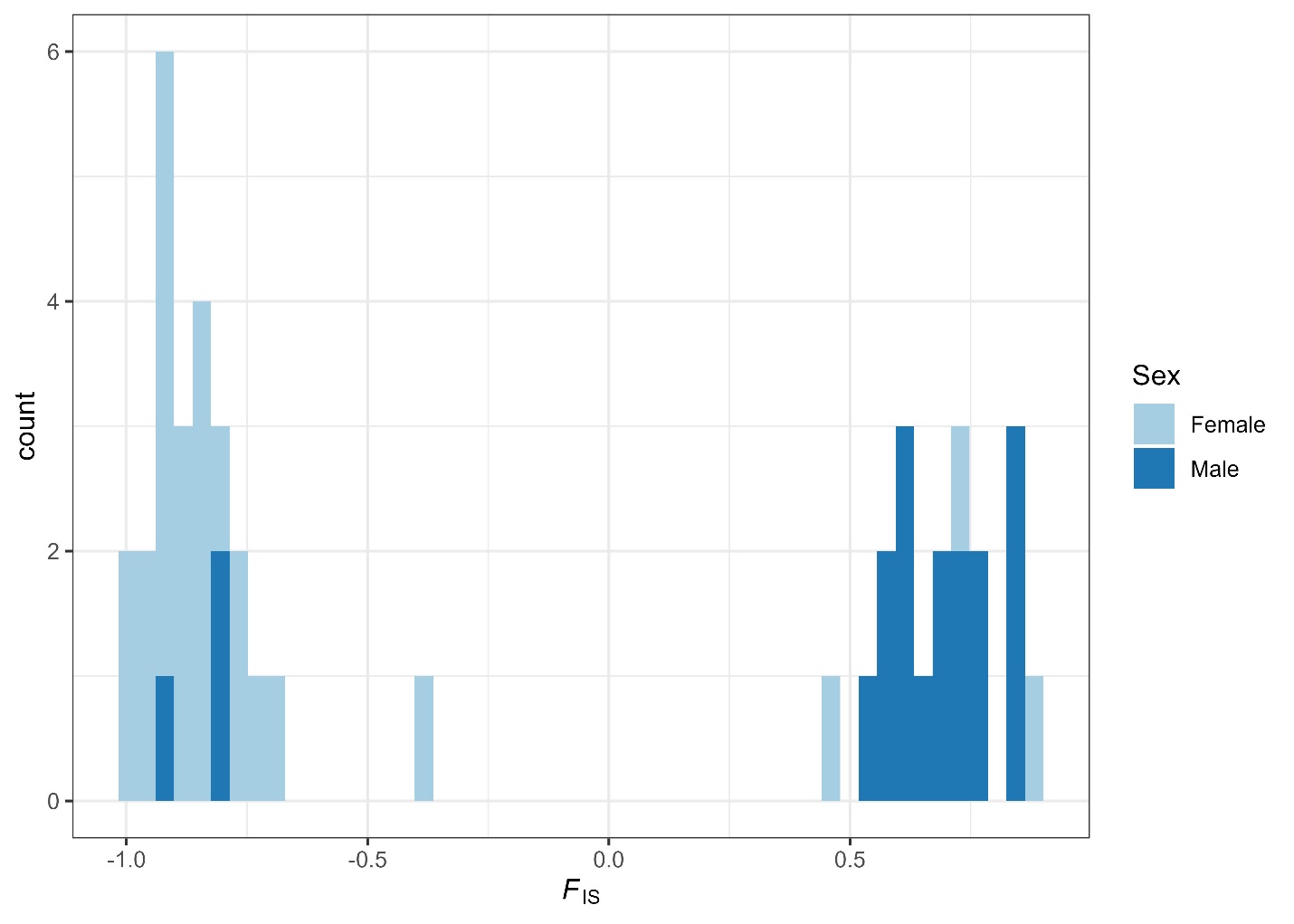
**

**Supplemental Figure 7 Histogram of *F*_IS_ in highly linked region of Chromosome 16 in Greenstriped Rockfish.** *F*_IS_ was calculated using *VCFtools*.


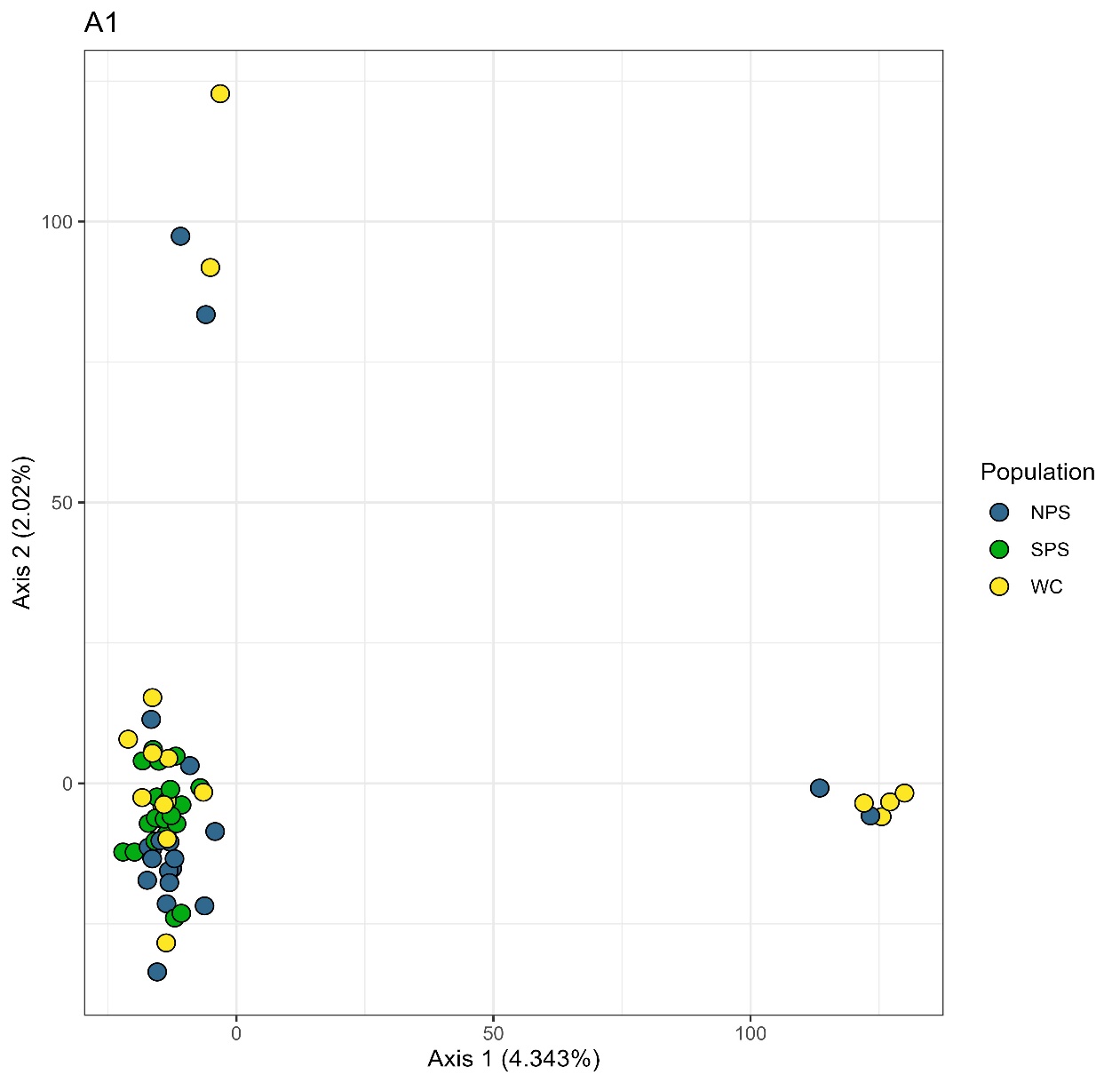


**Supplemental Figure 8 Principal components analysis of Black Rockfish including outlier individuals with very high homozygosity.** Each point represents an individual fish, colored by sampling location.

*
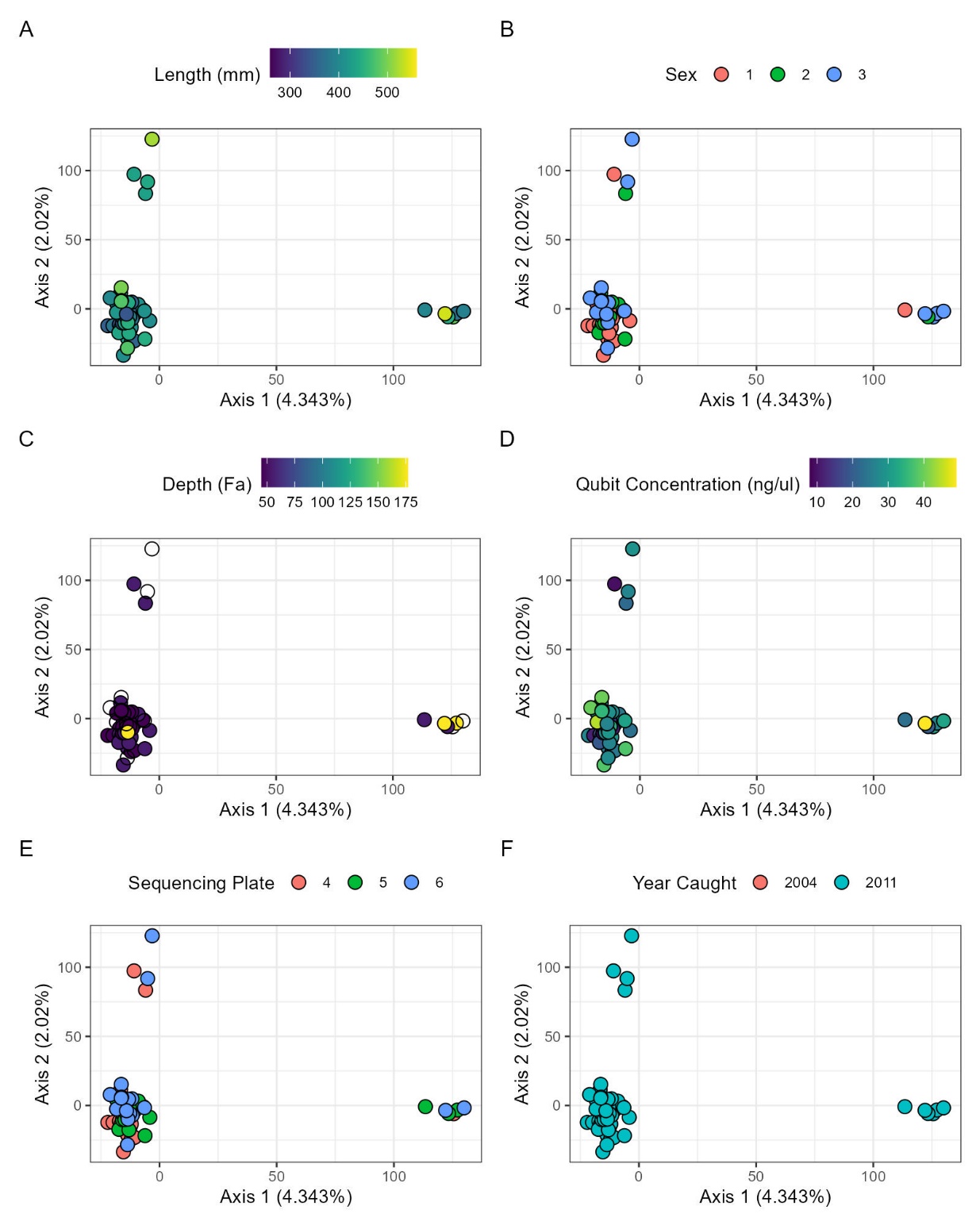
*

**Supplemental Figure 9 Principal components analysis of Black Rockfish labeled by life history characteristics.** Each point represents an individual fish, colored by various life history and methodology characteristics. Unfilled circles show missing data. A: length of fish (millimeters), B: sex (1 = Female, 2 = Male, 3 = Unknown), C: depth caught (fathoms), D: extraction Qubit concentration (nanograms per microliter), E: sequencing plate, F: year caught.


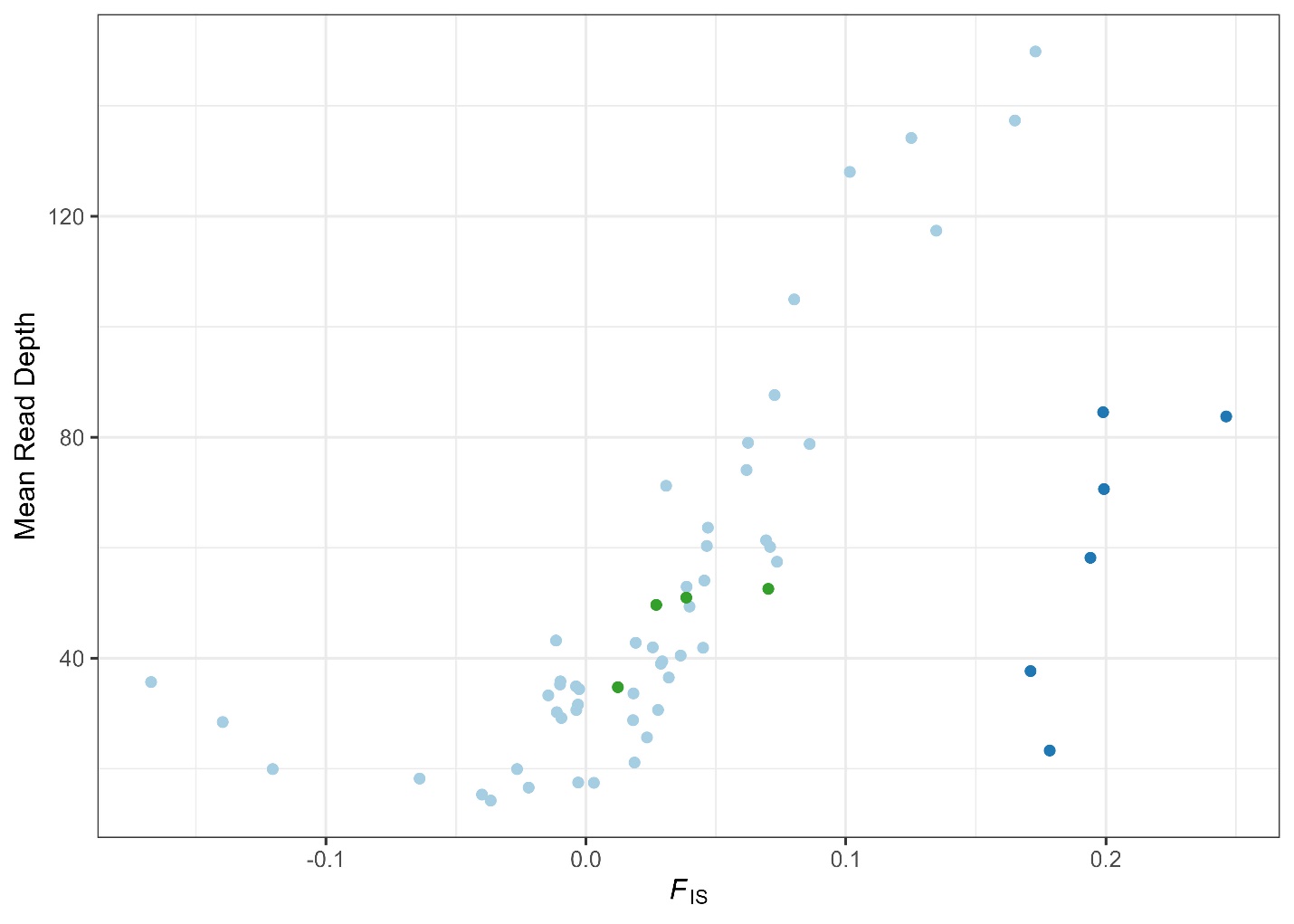


**Supplemental Figure 10 *F*_IS_ in Black Rockfish in relation to read depth.** Each point represents an individual fish. The outlying individuals removed from subsequent analyses are dark green and dark blue. Dark blue individuals show a high homozygosity compared to all other samples.

*
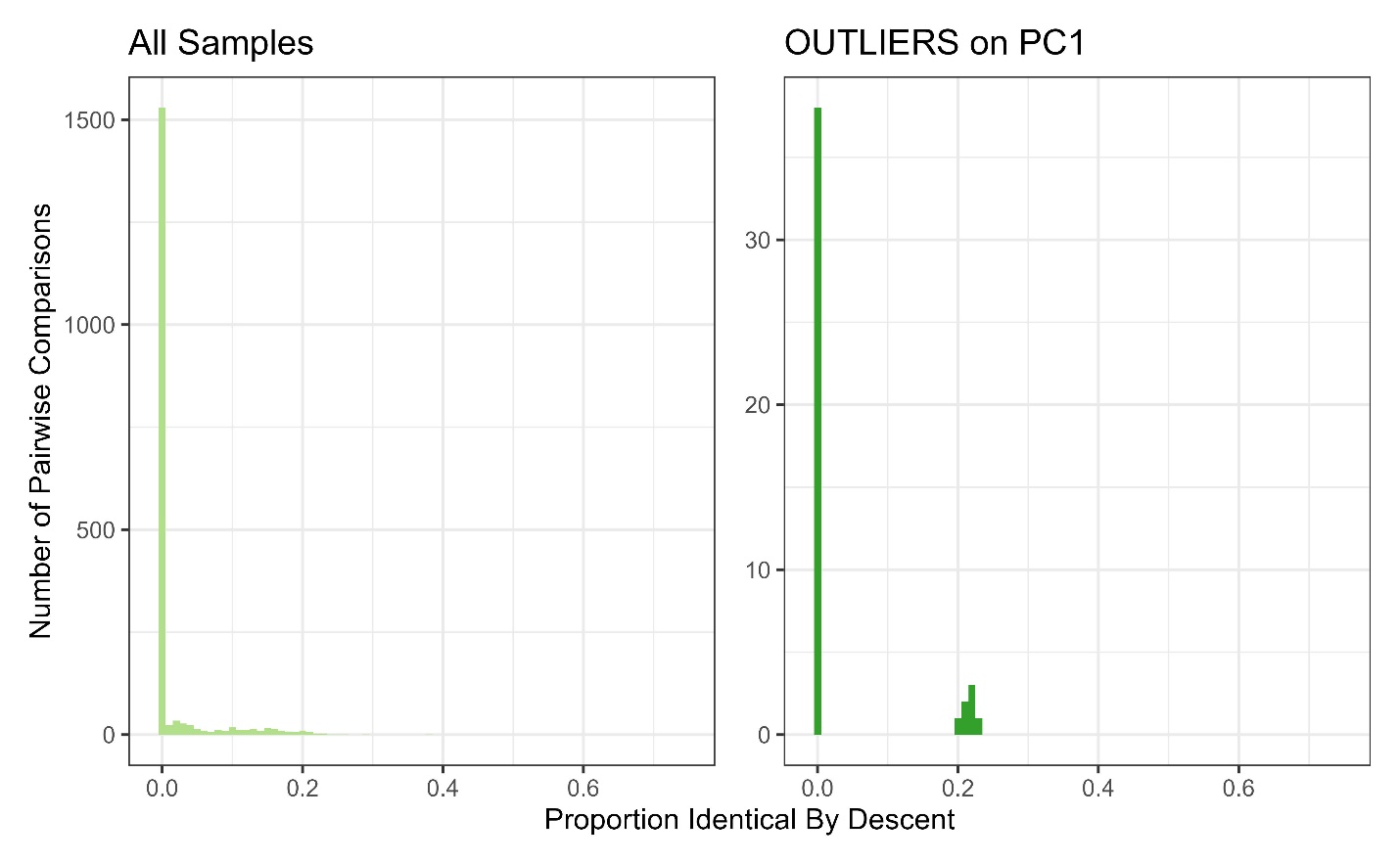
*

**Supplemental Figure 11 Pairwise relatedness estimates Black Rockfish individuals (left) and outliers (right).** Pairwise relatedness for the outlying individuals (right) was significantly different than in the admixed group (left, two-tailed t-test p-value = 0.011). Genetic relatedness was calculated using an identity by descent estimate on all pairs of individuals within *PLINK* v1.07 (Purcell et al., 2007).


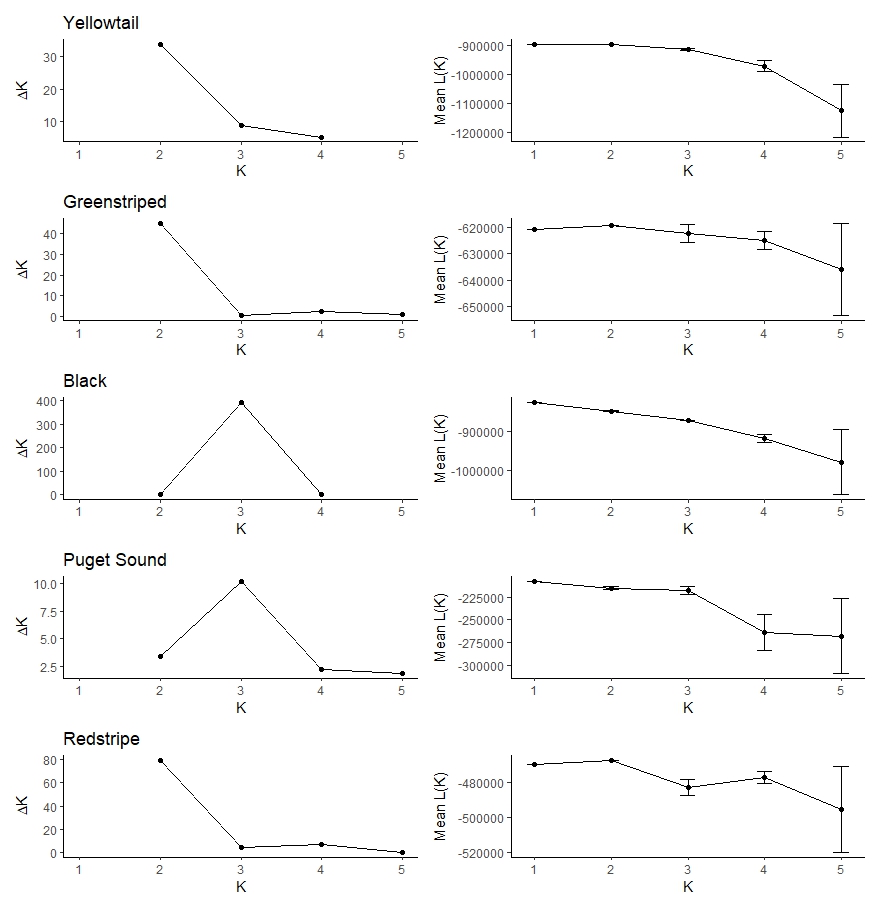


**Supplemental Figure 12 Mean likelihood and ΔK for *STRUCTURE* replicate runs for Yellowtail, Greenstriped, Black, Puget Sound and Redstripe Rockfish.** *STRUCTURE* was run without *a priori* population knowledge and using the admixture model. Two replicates were run for 1-10 clusters with a burn-in of 10,000 iterations and 100,000 MCMC reps.


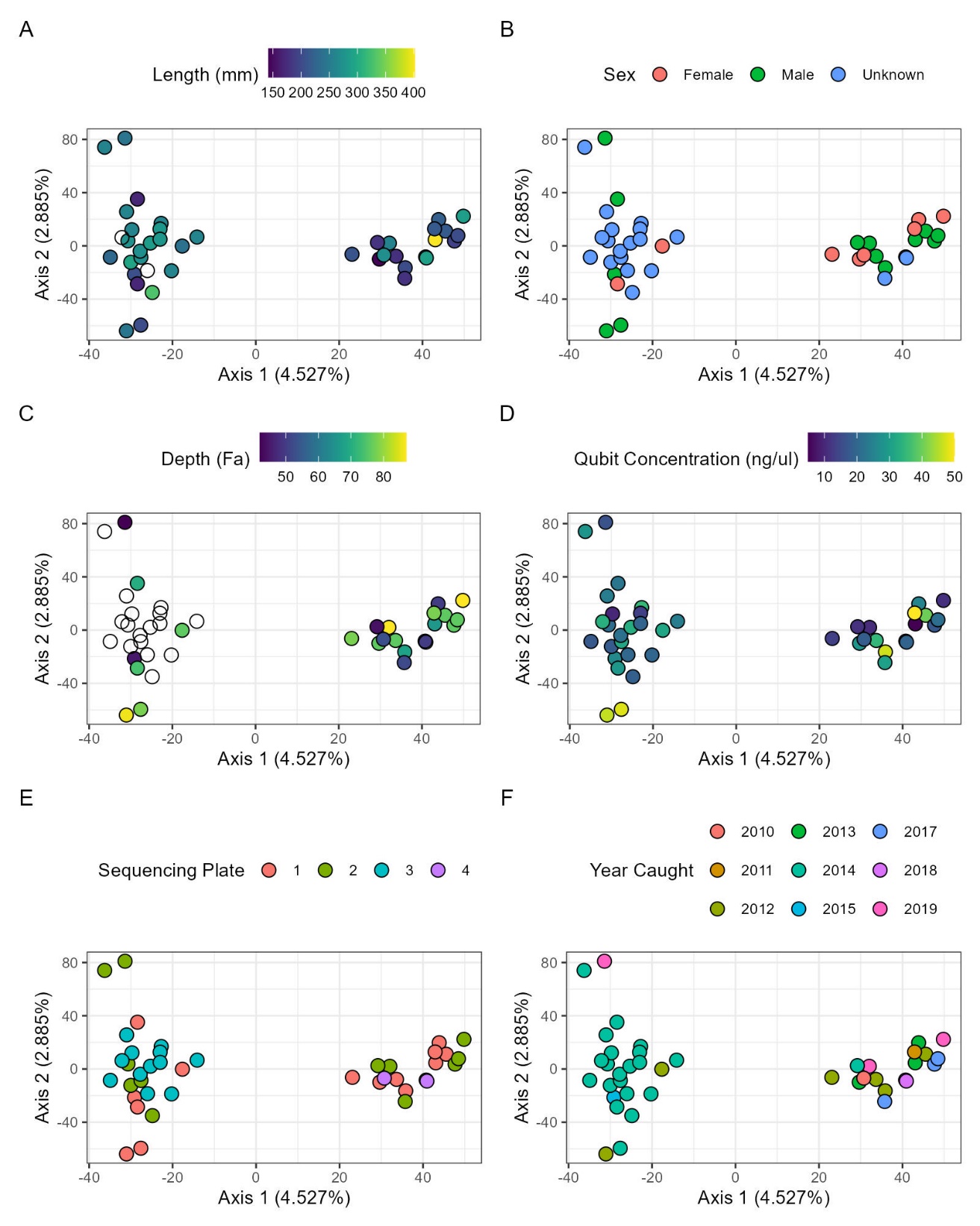


**Supplemental Figure 13 Principal components analysis of Redstripe Rockfish labeled by life history characteristics.** Each point represents an individual fish, colored by various life history and methodology characteristics. Unfilled circles show missing data. A: length of fish (millimeters), B: sex, C: depth caught (fathoms), D: extraction Qubit concentration, E: sequencing plate, F: collection year.


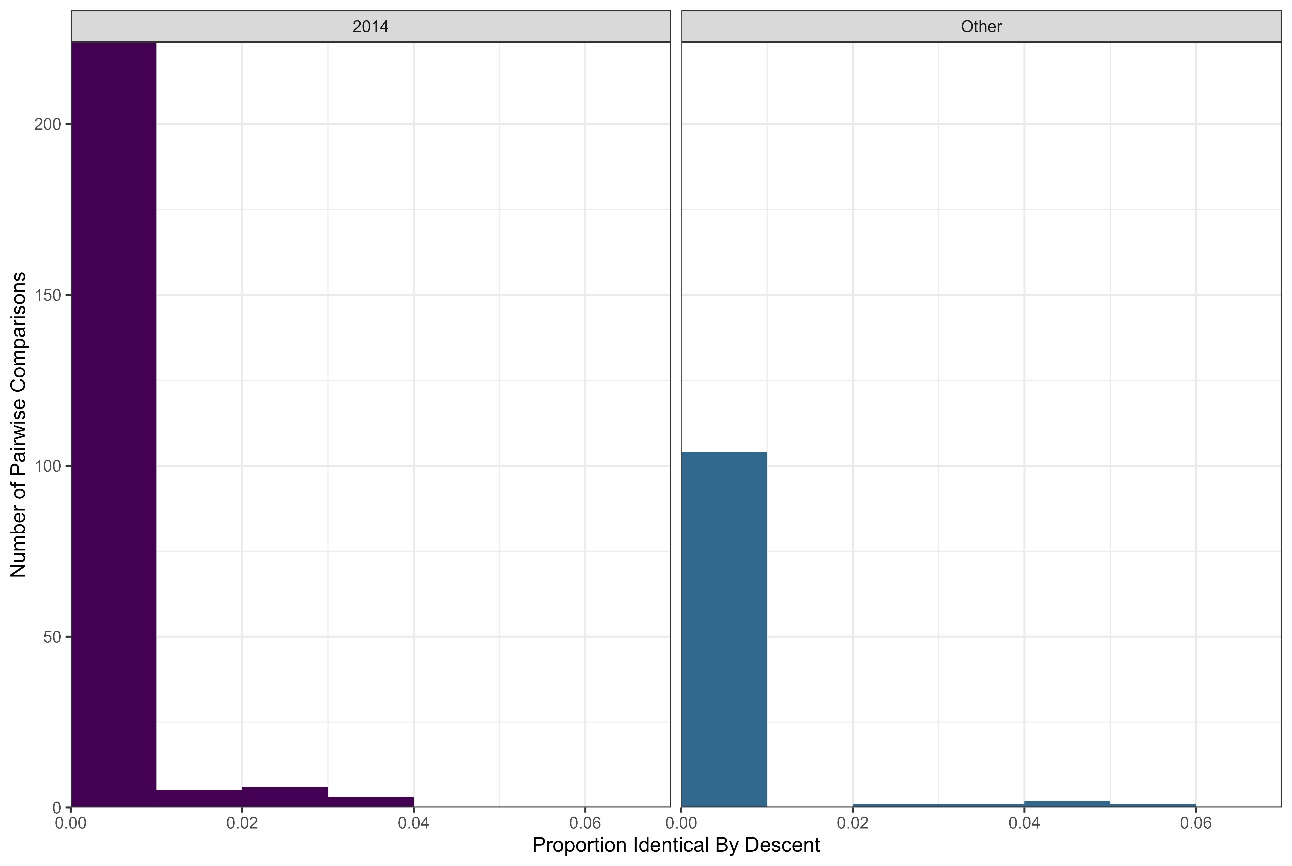


**Supplemental Figure 14 Pairwise relatedness estimates for 2014 Redstripe Rockfish individuals.** Pairwise relatedness for the 2014 individuals (left) was not significantly different than in any other sampling year (right, t-test p-value = 0.69). Genetic relatedness was calculated using an identity by descent estimate on all pairs of individuals within *PLINK* v1.07 (Purcell et al., 2007).


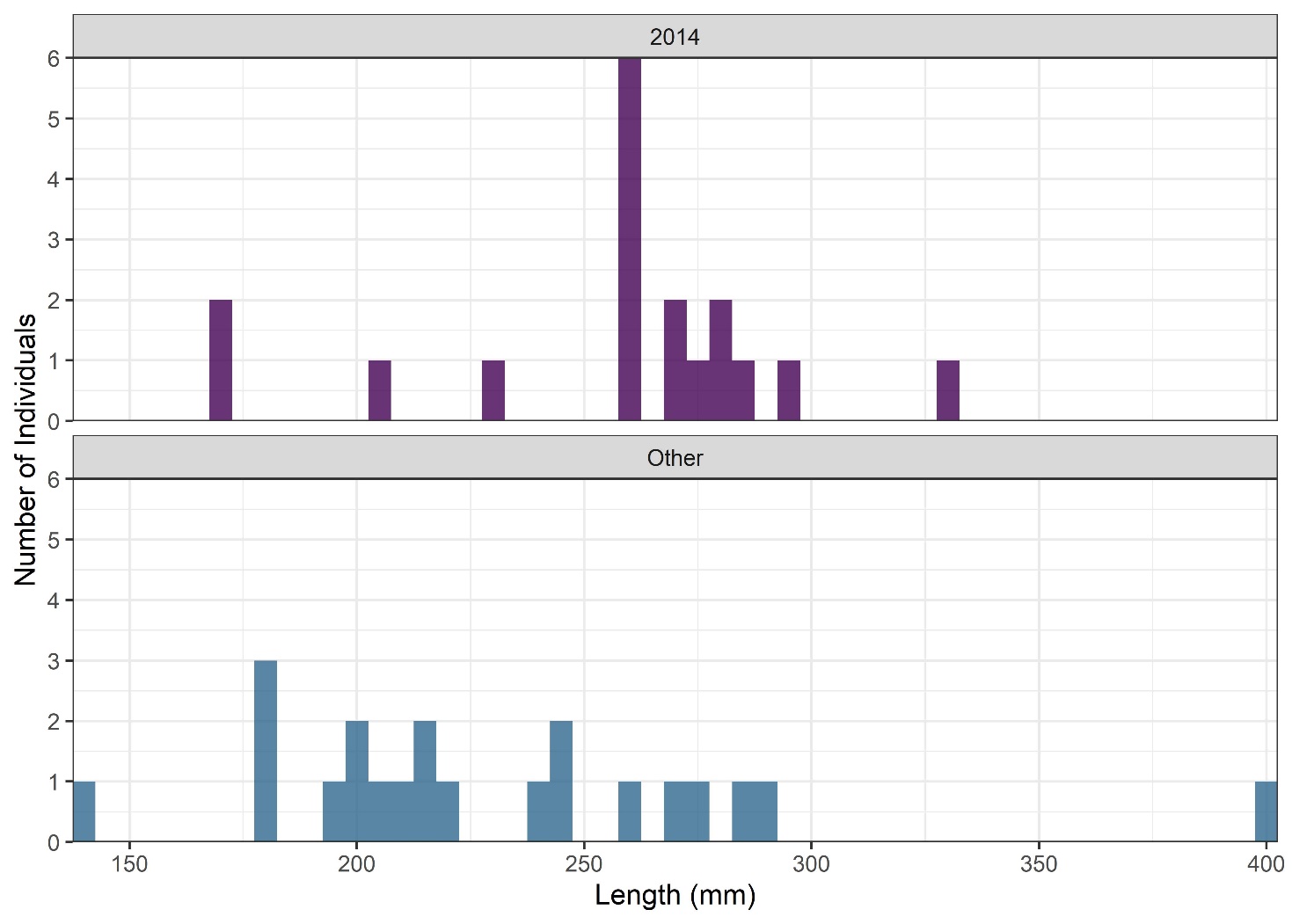


**Supplemental Figure 15 Distribution of fish lengths for 2014 Redstripe Rockfish individuals (top) compared to other sampling years (bottom).** Individuals collected in 2014 were larger on average than all other year classes combined (256 mm for 2014 and 231 mm for individuals collected in other years). The variance for the 2014 individuals versus the other sampling years is 1.9 times smaller.


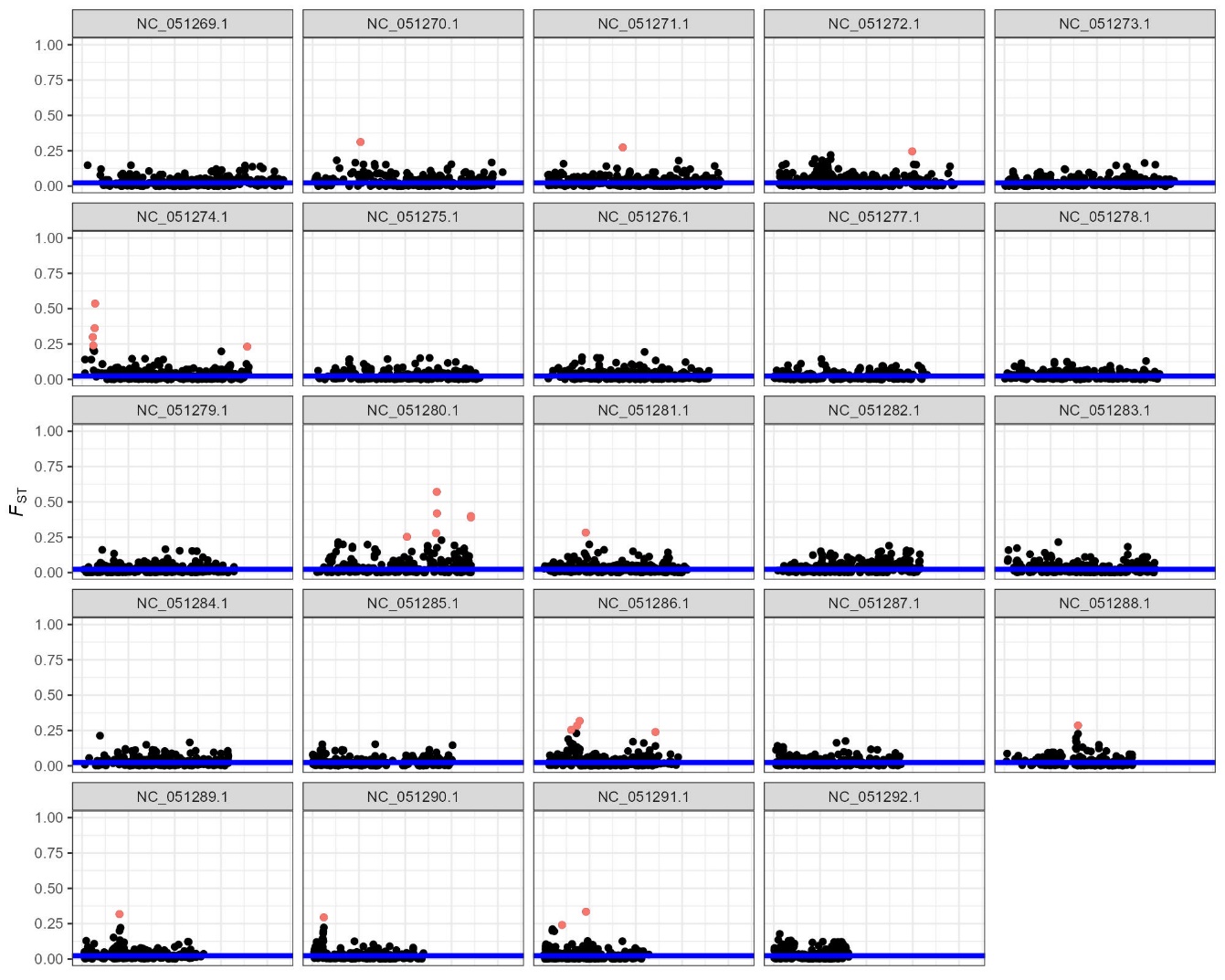


**Supplemental Figure 16 Manhattan *F*_ST_ Plot of two temporally divergent groups in Redstripe Rockfish.** *F*_ST_ was calculated using *hierfstat*. Overall *F*_ST_ between the two groups was 0.023 (0.0220-0.0249, range after 1,000 bootstrap iterations) and is displayed as the blue horizontal line. Points are highlighted if they were 10x greater than the overall average *F*_ST_ or 0.23.


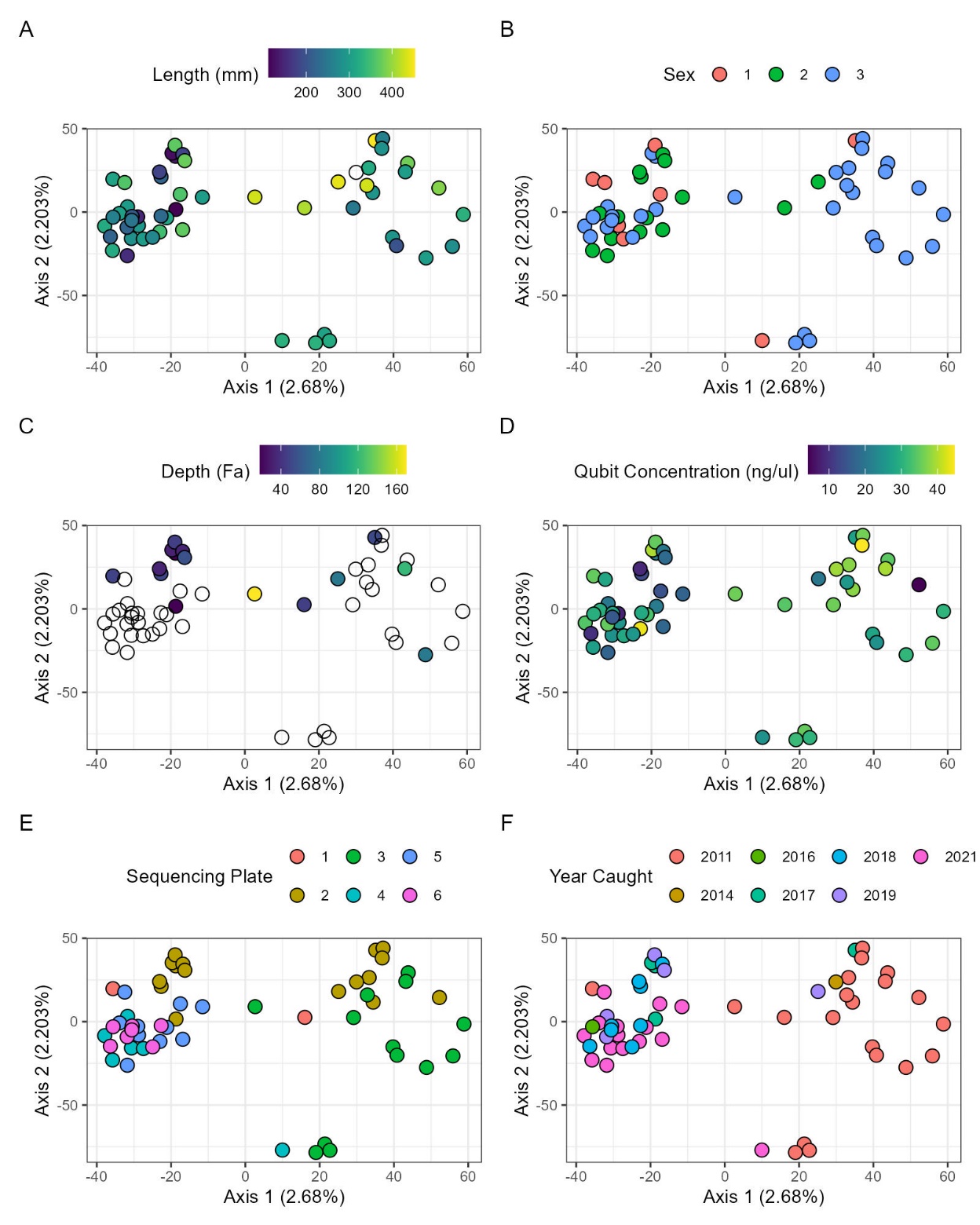


**Supplemental Figure 17 Principal components analysis of Yellowtail Rockfish labeled by life history characteristics.** Each point represents an individual fish, colored by various life history and methodology characteristics. Unfilled circles show missing data. A: length of fish (millimeters), B: sex (1 = Female, 2 = Male, 3 = Unknown), C: depth caught (fathoms), D: extraction Qubit concentration, E: sequencing plate, F: collection year.

**Supplemental Table 1 d_xy_ and divergence time estimation based on clustering pattern.** d_xy_ was calculated on all called SNPs and invariant sites using *pixy v1.2.7.beta1* (Korunes & Samuk, 2021) with a 10kb window. Mutation rate per generation was taken from Bergeron et al. (2023) and Zhang et al and was divided by the average generation time for each species (Kolora et al., 2023).

| **Species** | **d_xy_** | **d_a_** | **Mutation rate per year (λ)** | **Estimated Divergence Time** |
| --- | --- | --- | --- | --- |
| Yellowtail | 0.00169 | 0.0000112 | 2.94 x 10^-10^ | 18,978 |
| Redstripe | 0.00129 | 0.0000276 | 3.36 x 10^-10^ | 41,097 |
| Greenstriped | 0.00151 | 0.0000335 | 3.31 x 10^-10^ | 50,531 |

**Supplemental Table 2 N_e_ estimation based on clustering pattern.** Effective population size was calculated using the random mating, linkage disequilibrium model using *NeEstimator* with a MAF cutoff of 0.05 (Do et al., 2014) and adjusted for high loci number using Waples et al. (2016). Clusters were chosen based on PCA and *STRUCTURE* results. Parametric 95% confidence intervals are included in parentheses.

| **Species** | **Cluster** | **N_e_** |
| --- | --- | --- |
| Black | Outliers | ∞ |
|  | Admixed | 969 (2.7) |
| Yellowtail | Coast | 323 (1.8) |
|  | Puget Sound | 850 (1.7) |
| Redstripe | 2014 Year Class | 1,229 (4.4) |
|  | Other Year Classes | 729 (1.7) |
| Greenstriped | British Columbia | ∞ |
|  | Coast and Puget Sound | 1,141 (3.5) |
| Puget Sound | Admixed | ∞ |
